# Improving the understanding of sleep apnea characterization using Recurrence Quantification Analysis by defining overall acceptable values for the dimensionality of the system, the delay, and the distance threshold

**DOI:** 10.1371/journal.pone.0194462

**Published:** 2018-04-05

**Authors:** Sofía Martín-González, Juan L. Navarro-Mesa, Gabriel Juliá-Serdá, G. Marcelo Ramírez-Ávila, Antonio G. Ravelo-García

**Affiliations:** 1 Institute for Technological Development and Innovation in Communications, University of Las Palmas de Gran Canaria, Las Palmas de Gran Canaria, Spain; 2 Pulmonary Medicine Department, Hospital Universitario de Gran Canaria Dr. Negrín, Las Palmas de Gran Canaria, Spain; 3 Instituto de Investigaciones Físicas, Universidad Mayor de San Andrés, La Paz, Bolivia; Charité - Universitätsmedizin Berlin, GERMANY

## Abstract

Our contribution focuses on the characterization of sleep apnea from a cardiac rate point of view, using Recurrence Quantification Analysis (RQA), based on a Heart Rate Variability (HRV) feature selection process. Three parameters are crucial in RQA: those related to the embedding process (dimension and delay) and the threshold distance. There are no overall accepted parameters for the study of HRV using RQA in sleep apnea. We focus on finding an overall acceptable combination, sweeping a range of values for each of them simultaneously. Together with the commonly used RQA measures, we include features related to recurrence times, and features originating in the complex network theory. To the best of our knowledge, no author has used them all for sleep apnea previously. The best performing feature subset is entered into a Linear Discriminant classifier. The best results in the “Apnea-ECG Physionet database” and the “HuGCDN2014 database” are, according to the area under the receiver operating characteristic curve, 0.93 (Accuracy: 86.33%) and 0.86 (Accuracy: 84.18%), respectively. Our system outperforms, using a relatively small set of features, previously existing studies in the context of sleep apnea. We conclude that working with dimensions around 7–8 and delays about 4–5, and using for the threshold distance the Fixed Amount of Nearest Neighbours (FAN) method with 5% of neighbours, yield the best results. Therefore, we would recommend these reference values for future work when applying RQA to the analysis of HRV in sleep apnea. We also conclude that, together with the commonly used vertical and diagonal RQA measures, there are newly used features that contribute valuable information for apnea minutes discrimination. Therefore, they are especially interesting for characterization purposes. Using two different databases supports that the conclusions reached are potentially generalizable, and are not limited by database variability.

## Introduction

Obstructive Sleep Apnea (OSA) is a widespread sleep respiratory disorder, characterized by repetitive breathing pauses due to upper airway collapse during sleep. It can be considered a public health problem, not only because of its high prevalence, 4% in men and 2% in women [1], but also because of its major health implications [2–5]. These include daytime drowsiness, cardiovascular disorders, such as hypertension, stroke, and glucose metabolism abnormalities, all of them leading to increased mortality rates. Due to its relevance, there is a wide range of articles on sleep apnea characterization and detection, its implications in the cardiovascular system, and the relationship between sleep apnea and the autonomic nervous system (ANS) [6–18].

The criterion used to decide whether a patient suffers from OSA is the mean number of apneas per hour of sleep: Apnea-Hypopnea Index (AHI) [19]. In both apnea and hypopnea, there is a complete (apnea) or partial (hypoapnea) cessation of airflow for at least 10 seconds. Subjects with AHIs greater than 5 are OSA diagnosed and ranked according to the following: AHI ranging [5,15]: mild sleep apnea; AHI ranging [15, 30]: moderate sleep apnea; and AHI greater than 30: severe sleep apnea.

### Motivation and problem description

The gold standard for OSA diagnosis is polysomnography (PSG). It includes the recording of different physiological signals throughout the night at the hospital supervised by specialist staff. The process is inconvenient for the patient, time consuming and very expensive for the health care system. That is why several authors are making a major effort to create automatic sleep apnea screening methods based on a smaller number of physiological signals and portable systems, with the aim of reducing waiting lists. The ECG signal has turned out to be especially interesting for screening purposes as it is modulated by sleep, breathing and the ANS. Moreover, it can be easily recorded using wearable devices, in particular the single-lead ECG signal. Most of the methods proposed in the literature for sleep apnea detection using the ECG introduce features derived from the heart rate variability (HRV) and the ECG derived respiratory signal (EDR) [6–15,17–18,20].

The normal HRV is based on the autonomic neural regulation of the heart and the circulatory system, and changes in the HRV mirror the effects of different physiological factors modulating the normal heart rate [21]. HRV studies are performed using the RR series obtained from the ECG. It is constructed by measuring the delay between two consecutive R-peaks of the electrocardiogram. The sequence of consecutive delays forms the RR series.

The HRV provides valuable information about sleep apnea, due to its effects on heart rate regulation. Under normal circumstances, there are periodic variations in the RR series due to breathing phases (cardio-acceleration during inhalation and cardio-deceleration during exhalation) that are called respiratory sinus arrhythmia (RSA) [22]. This periodic information can be observed around 0.25 Hz. Moreover, there is a cardiorespiratory phase synchronization (CRPS) that defines the coupling between heart rhythm and respiration. Several authors have addressed this topic and have concluded that this synchronization may change according to diverse physiological conditions, such as different sleep stages or age [22–23], and that the “coupling direction” is from breathing to heartbeat. Bartsch et al. demonstrated that both RSA and CRPS represent different aspects of the cardiorespiratory interaction [22].

During sleep, HRV dynamics and complexity change [6,24–25]. In particular, the respiratory muscles try to overcome the obstruction of the upper airway during an OSA episode. If efforts are unsuccessful, the blood oxygen level decreases and consequently, muscle effort increases until an arousal takes place to reestablish normal breathing. This process leads to bradycardia, that starts after the air flow interruption and continues during the obstruction until the arousal event. At this moment normal respiration is reestablished, and the reflex tachycardia starts. Some authors have attributed this pattern of brady- and tachycardia to a parasympathetic control of heart rate during sleep, interrupted by sympathetic activation that ends with the arousal [24,26]. All this results in an increase of HRV [27], sympathetic overactivity [28], and a loss of complexity [27]. Additionally, frequency components appear around 0.02 Hz as a result of the brady/tachycardia patterns, called ‘cyclic variation of heart rate’ (CVHR), that occur due to apnea repetition [6,29–30].

Since the underlying cardiorespiratory system and, by extension, the HRV, is dynamic, nonlinear, and nonstationary [31–32], many authors have introduced nonlinear methods for its analysis [33–37]. They are especially suited for the analysis of complex autonomic and respiratory control mechanisms that interact in the regulation of the cardiac function to maintain homeostasis [38]. Webber and Zbilut [39] and Guzzeti et al. [40] also suggested that the HR control system is a deterministic chaotic system modulated by the ANS, in which recurrences within a state space is one of its typical properties [39]. Recurrence is defined as the repeated occurrence of a given state of the system in time [41]. However, it is important to point out that there are also other authors, like Glass [42], who believe that the HRV does not display chaotic dynamics. This is considered a controversial topic that remains an open and widely discussed issue [42].

All these characteristics lead us to Recurrence Quantification Analysis (RQA), introduced by Zbilut and Webber in 1992 [43]. RQA is the quantitative analysis of Recurrence Plots (RPs), which represent the recurrences in a dynamical system. Analyzing RP structures allow us to obtain information about the system dynamics. Compared with other nonlinear analysis methods, this technique is suitable for the analysis of short and non-stationary data, or when the system dynamics is located in a higher dimensional space, like the cardiac system [38,44]. A notable characteristic of RQA is that, unlike other techniques, no data transformation is needed. It is only necessary to represent similar events within an embedded space [45].

Despite RQA's advantages in the detection of dynamic changes, it is important to point out that, as relevant researchers in the matter state [45–48], results can be easily influenced by the setup parameter values, mainly the embedding parameters (delay and dimension) and the distance threshold, necessary for constructing the RP [46]. In general, the estimation of dynamical invariants is not dependent on the embedding, but the RQA measures are [47–48].

### Review of relevant literature

RPs were first introduced in 1990 by Zbilut et al. [49] for HRV analysis. Mammoliti et al. [50] applied RQA to derive information about nonlinear properties of HRV, employing RQA to inter-beat interval (RR) series. They stated that HRV in healthy human subjects in a relaxed state is characterized by the definite presence of complex and deterministic behavior. However, they also suggested that further studies would be necessary to compare results in other physiological conditions, such as illness. In the last 15 years, many authors have introduced RQA, among others, for the analysis of HRV in ventricular tachycardia [33], ventricular arrhythmias [32], sleep apnea [11–13,34], diabetes mellitus [41], paroxysmal atrial fibrillation [36], preeclampsia [51], and vasovagal syncopes [52]. So RPs and RQA can be considered a classical tool to analyse the cardiac system.

RQA was first introduced in sleep apnea studies by Maier and Dickhaus [34]. They compared the results obtained when using the distance matrix (DM), the RP and spectral techniques, applied to HRV, and concluded that spectral techniques, being simpler and computationally less demanding, yielded comparable results to those obtained with the other approaches. Therefore, they questioned the utility of RQA to yield additional insight into sleep apnea recognition from HRV. The selected values for embedding dimension and delay, obtained from preliminary research, were 7 and 5 respectively. As far as the distance threshold (*ε*) is concerned, they justified not using a fixed value, due to the considerable inter- and intra-individual variability. Instead, they controlled the number of recurrent points for each vector, assigning *ε*_j_ the 5th percentile of the distribution of all distances. On the other hand, they also worked directly with the recurrence matrix, thus avoiding the use of any threshold. It is important to emphasise that only measures based on diagonal structures were used in the experiments. The authors did not rule out the possibility that other RQA measures and another set of parameters may yield better results.

Le et al. [11] combined RQA features and power spectral density (PSD), obtained from the RR intervals, and employed SVM to determine the sleep apnea events. They used the same values as Maier and Dickhaus [34] for the embedding parameters (dimension: 7 and delay: 5), and for the distance threshold, 10% of the maximum phase space diameter. They introduced, beyond the typical diagonal and vertical lines related RQA measures, the recurrence times of 1st and 2nd type, the recurrence period entropy density, and the transitivity. According to their results, the most sensitive RQA feature is the length of the longest vertical line.

Karandikar et al. [12] applied RQA to HRV and to EDR signals and carried out different combinations to assess the classification system. Like Maier and Dickhaus [34], and Le et al. [11], they chose 7 and 5 for the embedding parameters, dimension and delay, respectively, and concluded, as Le et al. [11], that the most sensitive RQA feature was the length of the longest vertical line. There is no explicit reference to the method used for the distance threshold selection.

Nguyen et al. [13] applied RQA only to the HRV signal. They used the Fixed Amount of Nearest Neighbours (FAN) criterion for the distance threshold selection for the first time in the context of sleep apnea. Thus, they tried to better capture HR dynamics associated with OSA. Particularly, eight different values were tested, ranging from 2.5% to 20%, as they tried to extract a more comprehensive profile representation of the underlying dynamics. In their opinion, FAN is more suitable for HRV data analysis, since it does not require attractors to be of a similar volume for the comparison of state-space behaviors. In this way, a predefined percentage of recurrence points is set for all states [53]. They also changed the selection of the embedding parameters and chose for the dimension 6 and for the delay 10. This way they improved the results obtained in previous work. Unlike other authors, they found the embedding dimension by referring to the estimation of the number of representative variables expected to influence the system. Furthermore, the delay was determined as the time required to achieve variable independence and avoid redundancy. For classification purposes, they used SVM, neural network (NN), and a soft decision fusion rule to combine their results.

And finally, Cheng et al. [18] applied a modified version of RQA, heterogeneous RQA (HRQA) to sleep apnea, and again changed the values for the embedding parameters. They chose 5 and 3 for delay and dimension, respectively.

Analyzing the studies carried out by different authors using RQA applied to HRV (see Table 1) [32–33,36,41,50–56], and, in particular, in the context of sleep apnea [11–13,18,34], we can conclude that there is neither an agreement about the optimal set of parameters that should be used, nor about the best method to find the distance threshold. Most of the latter articles use the same database, the Apnea-ECG Physionet database, as well as the same strategies to obtain the parameter values. However, we find different figures for the dimension of the system, 3 [18], 6 [13] and 7 [11–12,34]; and for the delays, 5 [11–12,18,34] and 10 [13]. Different strategies are introduced for the threshold. The most important aspect is that the choice of these parameters is related to the structure and dynamic characteristics of the system underlying sleep apnea, and, although different values are found, they are all attempting to describe the same system.

**Table 1:** Selected parameters in articles where RQA is applied to HRV.

Article	Delay	Dimension
[50] RQA describes the complex and deterministic behavior of HRV in healthy subjects	4	8
[54] A nonlinear explanation of aging-induced changes in heartbeat dynamics	1	15
[33] Recurrence Quantification Analysis to characterize the heart rate variability before the onset of ventricular tachycardia	1	3–15
[55] Recurrence quantification analysis as a tool for nonlinear exploration of nonstationary cardiac signals	-	10
[56] Recurrence-plot-based measures of complexity and their application to heart-rate-variability data	-	3, 6, 9, 12
[32] Linear and nonlinear evaluation of ventricular arrhythmias	8	10
**[34] Recurrence analysis of nocturnal heart rate in sleep apnea patients**	**5**	**7**
[41] Recurrences in heart rate dynamics are changed in patients with diabetes mellitus.	Individually set for each recording	10
[53] The effect of orthostasis on recurrence quantification analysis of heart rate and blood pressure dynamics	Individually set for each recording	10
[36] Prediction of paroxysmal atrial fibrillation using recurrence plot-based features of the RR-interval signal.	1	7
**[11] Prediction of sleep apnea episodes from a wireless wearable multisensor suite.**	**5**	**7**
**[12] Detection of sleep apnea events via tracking nonlinear dynamic cardio-respiratory coupling from electrocardiogram signals.**	**5**	**7**
[51] Classifying healthy women and preeclamptic patients from cardiovascular data using recurrence and complex network methods.	-	-
**[13] An online sleep apnea detection method based on recurrence quantification analysis.**	**10**	**6**
[52] Recurrence plot of heart rate variability signal in patients with vasovagal syncopes.	-	-
**[18] Heterogeneous recurrence analysis of heartbeat dynamics for the identification of sleep apnea events.**	**5**	**3**

Articles related to sleep apnea are highlighted in bold.

‘-’ appears in the cases where no explicit value was given.

### Objectives of this study

Although many authors have analysed HRV in the context of apnea detection, the mechanisms are still unclear and, therefore, there is no defined model that describes the complex dynamics within the cardiovascular system during apnea. That is why we consider there is a margin for further studies to improve the characterization of the underlying process and to explore new features which extract as much information as possible from the ECG.

There are two main goals in this article. On the one hand, we concentrate on the selection of the parameters involved in RQA. As there are no overall accepted values for the study of HRV using RQA in sleep apnea, we carry out a thorough exploratory analysis of the system, sweeping the three most important parameters involved in RQA, dimension and delay for the embedding, and threshold selection for the RQA evaluation, simultaneously. Thus, we can also analyse the cross effects in the selection of the different parameters. The objective would be to find reference values for the different parameters implied in the RQA approach in the context of sleep apnea recognition from HRV, and to reach conclusions about the structure and dynamic characteristics of the underlying physiological system. On the other hand, we focus on furthering the knowledge of sleep apnea by uncovering the most relevant RQA features that best describe the RR pattern in OSA.The most representative features of the sleep apnea mechanisms are chosen using a forward feature selection algorithm.

The selected features are the input of a Linear Discriminant classifier, that produce a minute-by-minute classification of apnea and nonapnea minutes, also called quantification or per-segment classification. The experiments were carried out using two databases to give the results a more generalizable character, so that conclusions would not be limited by database variability.

## Materials and methods

### Databases

Two databases are used in the experiments, namely, the widely used Apnea-ECG Physionet database, referred to in the text as Physionet database, provided by Prof. Dr. Thomas Penzel for Computers in Cardiology Challenge 2000 [57], and the HuGCDN2014 database [58], provided by the Dr. Negrín University Hospital (Canary Islands, Spain). Neither of them distinguishes between apnea and hypopnea, defining them both as apnea.

The Physionet database consists of 70 single-lead ECG recordings, digitized at 100 Hz with 12-bit resolution. Their duration varies between 401 to 578 minutes (about 8 hours). Each minute was labeled as apneic or nonapneic by a human expert based on other signals recorded simultaneously.

According to the number of apneic minutes, the subjects were classified in three groups: 1º) GROUP A: recordings with at least 100 minutes with apnea (fifteen men and one woman). 2º) GROUP B: recordings with a number of apnea minutes between 5 and 99 (four men and one woman). 3º) GROUP C: recordings with fewer than 5 minutes with apnea (six male and five female subjects). Both the learning set (L) and the test set (T) are made up of 20 class A recordings, 5 class B recordings, and 10 class C recordings.

The HuGCDN2014 database was provided by the sleep unit of the Dr. Negrín University Hospital. It is made up of 77 single-lead ECG recordings, digitized at 200 Hz. The labeling process was performed by an expert based on the simultaneous polysomnography, indicating the presence or absence of apnea in each minute. The database is divided into two groups: 1º) CONTROL: Forty healthy subjects with an AHI lower than 5 (30 men and 10 women). 2º) APNEA: Thirty-seven OSA patients with an AHI higher than 25 (30 men and 7 women). The learning set (L) consists of 20 recordings of control subjects and 18 OSA patients. The rest belong to the test set (T).

### Preprocessing of the signal

The single-lead ECG signal is divided into 5-minute frames, that are shifted in time in increments of 1 minute. The RQA measures and the quantification obtained for each segment are assigned to the minute located in the middle position. Despite the fact that the analysis is done on a minute-by-minute basis, 5-minute frames are also suitable since a CVHR oscillation varies between 20 and 60 s, so recurrence of CVHR is only recognizable if several oscillations are contained in the frame [34]. Le et al. [11] use a larger window (10 minute windows and 1 minute sliding step), that in our opinion is not necessary.

The R-peak detection is inspired by the Pan-Tompkins algorithm [59], and the RR interval series is constructed as a sequence of time differences between the successive heartbeats. Once the RR series are obtained, an adaptive filtering procedure for automatic artefact removal is applied [60]. This is necessary as artefacts and ectopic values often corrupt HRV analysis. The advantage of this method is the spontaneous adjustment of the system coefficients to sudden changes in the series.

### Recurrence plots and recurrence quantification analysis

Recurrence, first introduced by Poincaré in 1890 [61], is a fundamental property of deterministic dynamical systems [33]. Eckmann et al. proposed in 1987 the use of RPs to visualize the recurrence characteristics of systems [62]. An RP is a two-dimensional plot that represents a binary symmetric square recurrence matrix. This defines the times where two states can be considered neighbours in the phase space, as they are in close proximity, according to a cut-off threshold [36,63]. That is, it allows us to visualize recurrences of a trajectory in a phase space and can be useful to uncover hidden periodicities and characteristics which otherwise would remain unnoticed [45].

Before constructing an RP of a time series, *u(t)*, data must be embedded in a phase space, as nonlinear data analysis is based on the study of the time evolution of a dynamical system in a given phase space [64]. The goal is to reconstruct a multivariate phase space that represents the original system. The most widely used strategy is Takens time delay method [65]. It is based on the fact that a higher-dimensional system, consisting of multiple coupled variables, can be reconstructed from a single measured variable of that system [63]. It is a very valuable method, since it allows us to study the system dynamics registering only one of its variables. The reconstruction is carried out generating time-delayed copies of the variable under study. In this way, the original time series is considered one dimension of the underlying system and each of its delayed copies becomes a new dimension of the system. The elements of the constructed phase space represent possible states of the structure. A phase state is defined as follows:

$${\vec{x}}_{i}=[u(i),u(i+\tau ),\ldots ,u(i+(m-1)\tau ]$$ (1)

where *m* is the embedding dimension and *τ*, the time delay. The dimension *m* is estimated taking into account the number of independent variables influencing the system under study. Physiological systems are controlled by a large number of continuously changing and interacting variables accompanied by noise [55]. These networked interactions take place according to couplings and feedback mechanisms that occur at multiple levels [66–68]. Finding out the optimal value for the dimension through the exploratory analysis will help us to infer the number of variables that underlie sleep apnea in the cardiovascular system. The greater the number of variables, the more complex the system [63]. So, if we consider that *m* components can represent a state at a certain time *t*, we will assume an *m*-dimensional phase space. It is important to take into account that, for noisy or random data, higher dimensions may be necessary [69]. The delay is chosen so as to achieve variable independence, avoiding the construction of state vectors that are autocorrelated [70].

After the embedding, the RP is created according to the following equation:

$$R_{i,j}=\Theta (\varepsilon_{i}-\|{\vec{x}}_{i}-{\vec{x}}_{j}\|),\;i,j=1,2,\ldots ,N$$ (2)

where *N* is the number of reconstructed points ${\vec{x}}_{i}$, *ε*, the threshold distance, Θ, the Heaviside function (Θ(*x*) = 0 if *x* < 0 and Θ(*x*) = 1, otherwise), and ||·||, the norm [45]. First, the distance matrix (DM) is constructed, and afterwards, the cutoff distance is applied to find the recurrence matrix (RM). In this way we obtain an N x N symmetric matrix, containing *R*_*i*,*j*_ = 1, if ${\vec{x}}_{i}$ and ${\vec{x}}_{j}$ are neighbours, according to the *ε-*threshold, and *R*_*i*,*j*_ = 0, if not. The RP is the graphical representation of the RM. As *R*_*i*,*i*_ = 1, the RP always has a black main diagonal, called line of identity (LOI). Elements near the main diagonal correspond to short-range correlations and distant ones to long-range correlations [54]. In case of stochastic or chaotic signals, RPs are formed by isolated points with no, or very short, diagonal structures, whereas periodic and deterministic signals show longer diagonals with less single recurrence points [36]. However, it is important to take into account that only periodic signals and white noise can be identified with some confidence. For the remaining signals, observing the RP structures does not let us reach definite conclusions about the system dynamics [48].

Since the selection of *ε* is decisive for the results, it must be chosen carefully. If *ε* is chosen too small, the different points of the trajectory will have hardly any neighbours, resulting in very few recurrences. However, if it is chosen too large, almost every point in the phase space will be considered a neighbour, leading to many artefacts [45]. Therefore, the *ε* selection is a trade-off, it should be chosen as small as possible, but large enough to have sufficient recurrence structures to quantify. Noise is another aspect that can influence the choice of the threshold, as it can make larger values necessary.

The commonly used RQA features are based on the recurrence point density and on the diagonal, vertical and horizontal line structures that appear in the RP [45]. There is another group of features that can be derived from RPs, which are related to recurrence times [45]. And finally, we also include new measures found in complex network theory, such as clustering coefficient or transitivity [71], that, when applied to recurrence matrices, are more powerful and reliable for the detection of periodic dynamics [48,72–74].

In this study, 17 features were extracted from the RP of each 5-minute frame. The first one is related to the recurrence point density: Recurrence Rate (REC). It quantifies the percentage of recurrent points in an RP and represents the average number of neighbours each element of the phase space has in its neighbourhood. Higher recurrence means lower system variability, since REC represents the probability that a certain state recurs [52].

$$REC=\frac{1}{N^{2}}\sum_{i,j=1}^{N}R_{i,j}$$ (3)

where *N* is the dimension of the recurrence matrix.

There is another group of features related to the diagonal lines structures: determinism, average diagonal line length, length of the longest diagonal line, and entropy. Diagonal lines appear in the RP in the case of parallel running trajectory segments [48]. Thus, a diagonal line represents a stable recurrence for a period coinciding with the length of the diagonal [75]. Particularly, if a diagonal is of length *l*, it means that a section of the trajectory is rather close during *l* time steps to another section, but at a different time [45]. This group of measures is based on the probability distribution, *P(l)*, of the lengths *l* of the diagonal lines, estimated from the histogram. By establishing a minimal length (*l*_*min*_) for it to be considered a diagonal line, we are able to adjust the sensitivity of the measures. RPs with no diagonals are typical for stochastic signals, very short diagonal lines for chaotic ones, longer diagonals correspond to deterministic processes and very long diagonal lines for periodic signals [63].

Determinism (DET) is the percentage of recurrent points forming diagonal lines of at least length *l*_*min*_ to all recurrent points [32]. It can be considered a measure of predictability and regularity of the system dynamics over time [41,45].

$$DET=\frac{\sum_{l=l_{min}}^{N}lP(l)}{\sum_{i,j}^{N}R_{i,j}}$$ (4)

It is common to define *l*_*min*_ = 2, since higher values could result in a sparse histogram, thus decreasing the DET reliability. In our studies, we define *l*_*min*_ = 2.

The average diagonal line length (L) is the average time that two sections of the trajectory are in close proximity and can be interpreted as the mean prediction time [45].

$$L=\frac{\sum_{l=l_{min}}^{N}lP(l)}{\sum_{l=l_{min}}^{N}P(l)}$$ (5)

The divergence is the inverse of the length of the longest diagonal line (Lmax). These measures are linked with the divergence of the phase space trajectory, i.e. shorter diagonal lines appear when trajectory sections diverge fast [45].

The entropy (ENTR) is the Shannon entropy of the diagonal lines length distribution.

$$ENTR=-\sum_{l=l_{min}}^{N}p(l)\;lnp(l)$$ (6)

where *p*(*l*) = *P*(*l*) / Σ_*l ≥ lmin*_ *P*(*l*). So, DET is concerned with the number of diagonals and ENTR with the distribution of the diagonal lengths.

DET, L and Lmax show higher values for more regular and correlated systems than for stochastic ones.

Another group of features is related to the vertical lines structures: laminarity, trapping time, and maximal length of vertical lines. Analogous to the diagonal lines structures, these features are based on the probability distribution, *P(v)*, of the lengths *v* of the vertical lines in the RP, considered only if they are longer than *v*_*min*_. As for *l*_*min*_, 2 is a commonly used value for *v*_*min*_, and we use it in our studies. Horizontal and vertical lines appear when a system state does not change for some time or changes very slowly. Therefore, they can be considered useful for the study of intermittencies. In general, RQA measures based on vertical structures are much more sensitive to the embedding than those based on diagonal ones [56].

The laminarity (LAM) is, as in DET, but for the vertical lines, the percentage of recurrent points forming vertical lines of at least length *v*_*min*_ to all recurrent points.

$$LAM=\frac{\sum_{v=v_{min}}^{N}vP(v)}{\sum_{i,j}^{N}R_{i,j}}$$ (7)

The trapping Time (TT) is the average length of vertical lines, i.e. analogous to L, but for the vertical lines.

$$TT=\frac{\sum_{v=v_{min}}^{N}vP(v)}{\sum_{v=v_{min}}^{N}P(v)}$$ (8)

TT estimates the average time the system stays in a specific state and contains information about the frequency of the laminar states and their lengths. High TT values represent systems consisting mainly of laminar states, whereas low TT values indicate systems without laminar states [45].

The maximal length of vertical lines (Vmax) gives information about the duration of the laminar states [45]. LAM is more robust against noise than TT and Vmax [52].

In the analysis of results, it is important to take into consideration that LAM, TT and Vmax are inversely proportional to the system complexity. This means that low LAM, TT and Vmax values imply high complexity in the system dynamics, because the system remains briefly in a state similar to the previously occurring one [41].

The other group of features is related to recurrence times: recurrence time type 1, recurrence time type 2, mean recurrence time, recurrence time density entropy, maximal recurrence time, minimal recurrence frequency, and entropy of the white vertical lines. Once the recurrence points are known, the recurrence times between them can be calculated. The recurrence times of type 1 (T1) and 2 (T2) are the average value of all recurrence times. The difference is that, for T2, the recurrence points are ruled out, due to possible tangential motion, i.e. T2 contains information about the time distance between the beginning of subsequent recurrence structures [45].

Recurrence time type 1 (T1) [76] is:

$$T1=\frac{1}{N}\sum_{i=1}^{N}T_{i}^{\left(1\right)}$$ (9)

$T_{i}^{\left(1\right)}$: the average of the minimum time difference between points in the neighbourhood of a point *i* on the reconstructed trajectory [77].

Recurrence time type 2 (T2) [76] is:

$$T2=\frac{1}{N}\sum_{i=1}^{N}T_{i}^{\left(2\right)}$$ (10)

$T_{i}^{\left(2\right)}$: the average return time (i.e. the minimum time difference between the recurrence points in the neighbourhood of point *i* on the reconstructed trajectory excluding all successive time points) [77].

The Mean Recurrence Time (RT) [78–79] is an alternative estimator for T2 but for the calculation, the focus is put on the white vertical lines [45]. Vertical and horizontal white bands result from rarely occurring states [80]. It is defined as the average of the lengths of the white vertical lines in the RP.

$$RT=\frac{\sum_{w=1}^{N}wP(w)}{\sum_{w=1}^{N}P(w)}$$ (11)

where *P(w)* stands for the frequency distribution of the lengths *w* of white vertical lines.

The Recurrence Period Density Entropy (RPDE) contains information about the periodicity characteristics of a signal in the context of dynamical systems. This measure is particularly suitable to detect repetitions of the same sequence of a time series in the phase space of the system.

$$RPDE={lim}_{\varepsilon \to \infty }{lim}_{m\to \infty }\frac{1}{\tau }ln\frac{c^{m}(\varepsilon )}{c^{m+1}(\varepsilon )}$$ (12)

The white vertical lines also indicate the maximal recurrence time (RTmax), as the longest length of the white vertical lines, the minimal recurrence frequency (RF), as the inverse of RTmax, and the entropy of the white vertical lines (ENTW).

And finally, there is a group of measures originating in the complex network theory. Marwan et al. [72] introduced in 2009 a new approach for analyzing time series using complex network theory by identifying the recurrence matrix with the adjacency matrix of a complex network, that represents the links between the nodes of the network. This equivalence is valid for undirected and unweighted networks. In this analogy, phase space vectors can be considered the nodes of a network, and the recurrences in the phase space, the links between them. Therefore, complex network measures can be applied on RPs in order to quantify the RP structure and the topology of the phase space. In this way, additional information can be obtained about the dynamics of the underlying process. The measures used in this article are transitivity and clustering coefficient. Transitivity of a complex network is related to the probability that two neighbours of any state are also neighbours, and this measure indicates how much a network is locally clustered [72,81]. Watts and Strogats [82] define another way of finding the local clustering degree, the clustering coefficient. First they calculate the local clustering coefficient for each node, and the clustering coefficient is the average of all nodes [81]. The adjacency matrix *A* used in their definitions is the recurrence matrix from which the identity matrix is subtracted (*A*_ij_ = *R*_ij_−*δ*_ij_ where *δ*_ij_ is the Kronecker delta) [79].

The clustering coefficient [72] is:

$$Clust=\sum_{i=1}^{N}\frac{\sum_{j,k=1}^{N}A_{i,j}A_{j,k}A_{k,i}}{{RR}_{i}}$$ (13)

where ${RR}_{i}=\sum_{j=1}^{N}A_{i,j}$ is the local recurrence rate.

The transitivity [79] is:

$$Trans=\frac{\sum_{i,j,k=1}^{N}A_{jk}A_{ij}A_{ik}}{\sum_{i,j,k=1}^{N}A_{ij}A_{ik}}$$ (14)

The CRP Toolbox (provided by TOCSY: http://tocsy.agnld.uni-potsdam.de) was used for the experiments.

### Parameter selection

Since the signal under analysis is dynamic, nonlinear and nonstationary, the choice of the different parameters that are necessary for the RQA analysis is not straightforward. However, regardless of the parameter values, it is important to guarantee that the features obtained from the datasets (representing apnea and nonapnea minutes) are calculated under the same conditions.

First, we focus on the selection of delay and dimension. The delay must be given before the minimum embedding dimension can be determined [83]. In general, there are two widely used methods to choose the delay: the (linear) autocorrelation (AC) or (nonlinear) mutual information (MI), calculating the first local minimum or the first zero crossing [63]. Webber and Zbilut [43,63] suggest setting the delay to 1 for RR signals, i.e. no points in the time series are skipped. As shown in Table 1, some authors have followed this suggestion [33,36], but others have proven different values [11,32,34].

**Fig 1:**
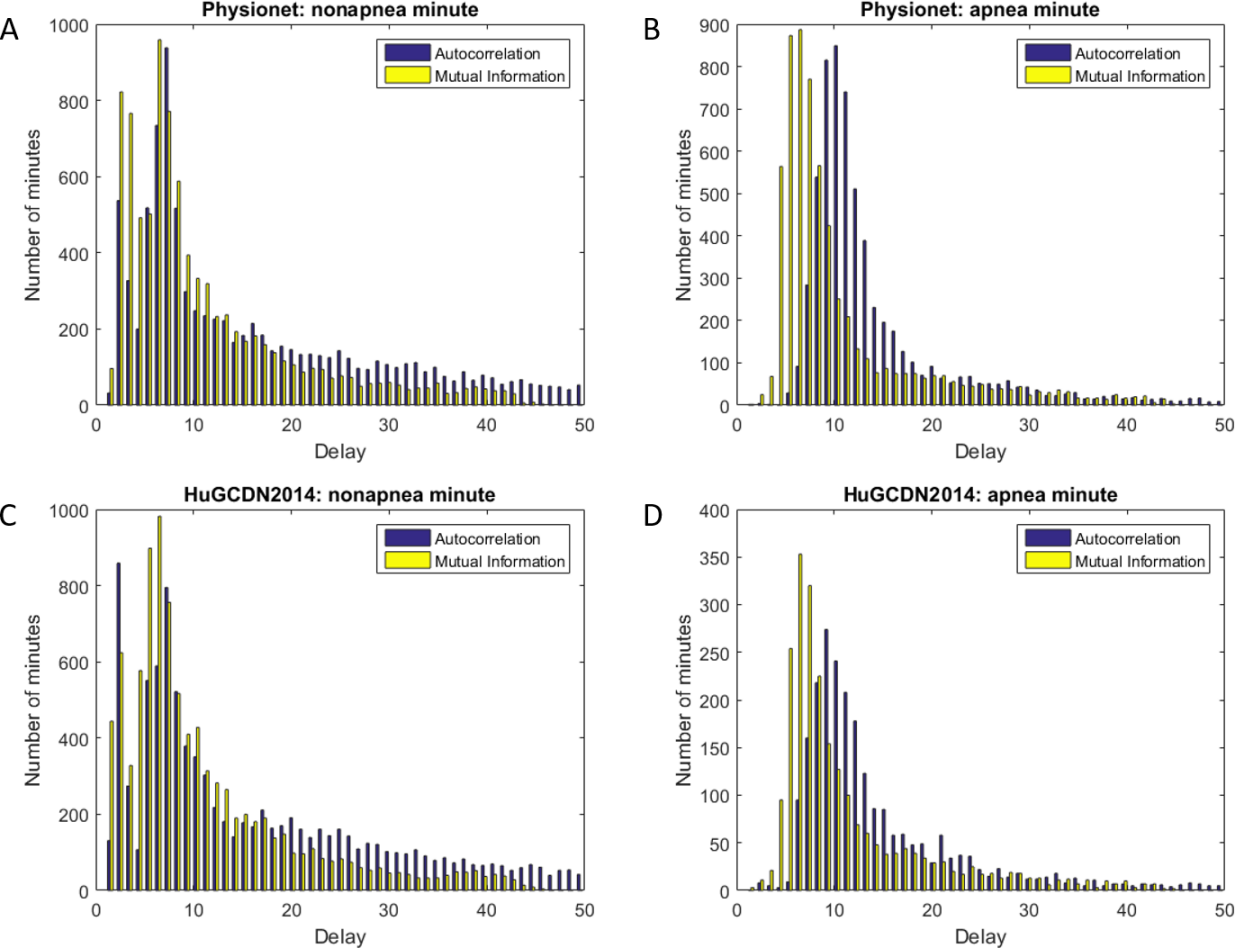
Optimal delays based on autocorrelation and mutual information for apnea and nonapnea minutes. (A and B) Physionet. (C and D) HuGCDN2014.

In the experiments the first zero crossing was considered. For each frame, representing a minute, the AC and MI were calculated. The results obtained for each of the databases, both for apnea and nonapnea minutes, are shown in Fig 1. The outcomes when using AC and MI are not exactly the same, especially in apnea minutes. Nevertheless, we can see a range, from 1 to 12, that is used in the experiments, where all maxima are included. This interval also contains the most common values used in literature for HRV analysis (see Table 1). If the delay is chosen properly, lower values for the minimum dimension may be necessary to reconstruct the phase space [83].

As far as the dimension is concerned, it is important to define a sufficiently large dimension that mirrors the relevant system dynamics. Choosing a dimension too low would cause points, that in the original phase space are far apart, to be considered closer in the reconstructed space [83]. The False Nearest Neighbours (FNN) Method is the most widely used to define the dimension. A neighbour is considered a false neighbour when it is viewed in a state space with a dimension that is too small. The dimension is increased in integer steps until the number of nearest neighbours becomes unchanged, i.e. when the number of false nearest neighbours drops to zero, meaning that the embedding of the time series is carried out in a proper dimensional space [36,84].

**Fig 2:**
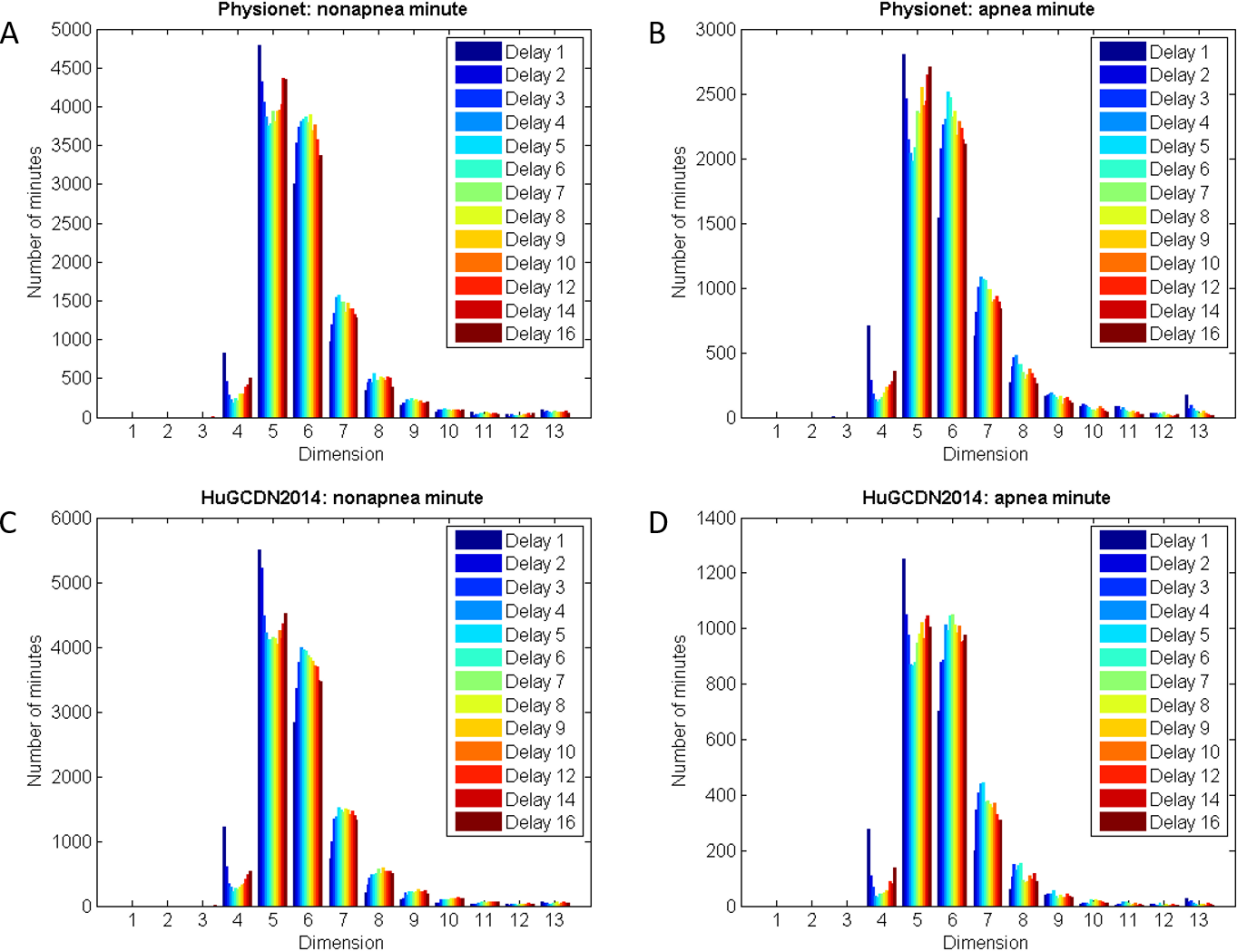
Dimension for apnea and nonapnea minutes according to different delays. (A and B) Physionet. (C and D) HuGCDN2014.

To define the dimension range in our experiments we used the FNN Method. Due to the practical dependence on the delay [83], delays ranging between 1 and 16 were employed to study the best values for the dimension. In agreement with the results shown in Fig 2, we can find in both databases the maxima for dimensions 5, 6 and 7, regardless of whether they are apnea or nonapnea minutes. As Cheng et al. [18] used dimension 3 for sleep apnea, we included dimensions 3 and 4 in our experiments. For the upper bound we chose 9. Seven is the highest dimension used in the context of sleep apnea but, in general, higher values are also found for HRV (Table 1). Therefore, we decided to include dimensions 8 and 9. In summary, for the dimension, we sweep the range from 3 to 9, and also include the possibility of non embedding (dimension = 1). In the latter case, RQA measures are directly obtained from the time series. This option was introduced by Ngamga et al. [79] in a study on epilepsy using EEG data.

It is important to take into account that HRV originates from the interaction of different control loops in the cardiovascular system and the ANS, leading to a timevarying phase space. So, optimal values for dimension and delay may change with time and, therefore, for every frame as well. In this respect, we can conclude that there is no optimal unique value for all patients and all minutes. However, according to the criteria followed when RQA is used for classification purposes, we should construct a common phase space with fixed values for delay and dimension, so that all the time series are embedded in the same phase space. We could use the average of the estimated dimension/delay values, but in our experiments the main goal is to extract information about the underlying physiological process. So we have swept a range of values for each of the embedding parameters (delay and dimension), and hence, we will be able to infer from the best results those values that best describe the system.

The other crucial parameter is the distance threshold. In general, we can either choose a fixed value, so that *ε*_*i*_ = *ε*, hereinafter referred to as Fixed Distance Method, or this parameter can be defined so that each point of the trajectory is surrounded by a constant number of neighbouring states, i.e. *ε*_*i*_ changes for each state, called Fixed Amount of Nearest Neighbours (FAN) Method. The latter case results in an asymmetric RP and a constant density of recurrence points in each of its columns [80]. In general, the Fixed Distance Method is used more often than the FAN Method. In the literature there are several works that address the optimal selection of the distance thereshold, *ε* [45–46,78,85–87], but, in spite of all the effort, it remains unsolved.

In the RQA analysis of HRV for sleep apnea detection, several authors have used the Fixed Distance Method and the FAN Method. In our experiments, we introduce both in order to evaluate which is more suitable in this context. In the Fixed Distance Method, the threshold is defined depending on the standard deviation of the particular frame, as this is one of the criteria especially suited for signal detection [78]. The selected values are based on the work done by Ramírez et al. [51]. For the FAN criterion, 12 different values were tested, ranging from 1% to 25%, based partially on the values chosen by Nguyen et al. [13]. However, their approach is different. They evaluate 9 RQA measures for eight different FAN values, obtaining 72 RQA features that are entered into a feature selection process. The final feature subset is made up of 33 features belonging to different FAN values. However, we assess the system performance for each of the FAN values, carrying out the feature selection process using the 17 features obtained for each of them.

We can see from all the previous work on the search for the necessary set of parameters involved in the relatively new RQA method, that it remains an open and widely discussed issue, especially in the case of the threshold distance.

### Classifiers

Since one of our goals is to extract as much information as possible from the physiological process associated with apnea, the classification process is done on a minute-by-minute basis, deciding whether the evaluated minute shows apnea or not. Linear Discriminant Analysis (LDA) is proposed for the quantification of apnea minutes, as this classifier balances performance, complexity and interpretation capacity.

The LDA classifier is based on a parametric model, whose parameters are adjusted using the learning set. We assume a class-dependent multivariate Gaussian distribution for the features:

$$f_{k}(x)=\frac{1}{{\left(2\pi \right)}^{\frac{p}{2}}{\left|\sum_{k}\right|}^{\frac{1}{2}}}e^{-\frac{1}{2}{\left(x-\mu_{k}\right)}^{T}\sum_{k}^{-1}(x-\mu_{k})}$$ (15)

where μ_k_ and Σ_k_ are the mean vector and covariance matrix of each class *k* (apnea and nonapnea). In LDA, the covariance matrices are considered equal in both classes (Σ_k_ = Σ).The parameters of the Gaussian distributions are obtained as follows:

$${\hat{\mu }}_{k}=\sum_{g_{i}=k}x_{i}/N_{k}$$ (16)

$$\hat{\sum }=\sum_{k=1}^{K}\sum_{g_{i}=k}(x_{i}-{\hat{\mu }}_{k}){\left(x_{i}-{\hat{\mu }}_{k}\right)}^{T}/(N-K)$$ (17)

One minute is classified as apnea if:

$$x^{T}{\hat{\sum }}^{-1}({\hat{\mu }}_{ap}-{\hat{\mu }}_{nap})>\frac{1}{2}\mu_{ap}^{T}{\hat{\sum }}^{-1}{\hat{\mu }}_{ap}-\frac{1}{2}\mu_{nap}^{T}{\hat{\sum }}^{-1}{\hat{\mu }}_{nap}+log\left(\frac{N_{nap}}{N}\right)-log\left(\frac{N_{ap}}{N}\right)$$ (18)

where ${\hat{\mu }}_{ap}$ and ${\hat{\mu }}_{nap}$ are the mean vectors of class apnea and nonapnea respectively, and *N_ap_* and *N_nap_* are the number of apnea and nonapnea observations.

Based on the estimation of the *a posteriori* probability for each class, the frame is assigned to the class that shows the largest value.

### Feature selection technique

A fundamental step after generating the feature vectors for each RR-frame, is the selection of the feature subset that best distinguishes between the two classes, since this selection facilitates the physiological interpretation of the results.

Therefore, we use a repeated random sub-sampling validation to reduce the dimensionality and, at the same time, increase the accuracy [16,20,30,37]. The selected features improve apnea quantification as they describe in greater detail the RR pattern in OSAS. We used 250 iterations in all cases under study, as this number allows us to find stable results.

In the process, we only use the learning set (L). We divide it into two equally-sized groups that form a training set and a validation set, each of them containing the feature vectors of the randomly selected patients in each iteration. In this way, we avoid feature vectors from one patient being simultaneously in the training set and in the test set.

The first step is to obtain a ranking of features created according to the number of times a certain feature is selected by the sequential forward feature selection method (based on the classifier performance). In each iteration, the optimal feature set corresponds to the maximum accuracy in the validation set.

In the second step, again repeating a random sub-sampling validation process 250 times, the error rate is obtained for an increasing number of features. They are entered in the same order as they appear in the ranking created in the first step. This allows us to analyze the evolution of the averaged misclassification error, obtained for the validation data, according to the number of features. The final selected features in the process will be those that produce the minimum misclassification error. In all cases, the number of features selected is smaller than the original number of features.

## Results

The general goal of all data analysis presented in this article is to detect the parameter combination that reaches the best discriminant capacity between apnea and nonapnea minutes when using RQA applied to sleep apnea. This allows us to infer information about the parameter values that best describe the HRV patterns associated to sleep apnea. On the other hand, the analysis of the selected features will allow us to detect those that are especially interesting for characterization purposes, as they show the highest discriminatory power.

Following Shinckel et al. [78], we use the area under the curve (AUC) of the receiver operating curve (ROC) as the main performance measure of the system, as it can be considered a summary of the ROC.

### Dimensions, delays, and distance thresholds

Tables 2–5 show the results (AUCs, accuracies, distance threshold, and number of selected variables) obtained for all combinations, using the test sets (T) of both databases. In each table, the best results for each dimension are highlighted. The first value in each box shows the AUC, the second one, the accuracy, and the fourth one, the number of selected variables. When the Fixed Distance Method is used for the threshold (*ε*) (Tables 2 and 4), the third value in each box represents the best value to multiply by *σ*, the frame standard deviation. When the FAN Method is used (Tables 3 and 5), the third value shows the percentage of neighbours to be considered.

**Table 2:** AUCs, accuracies, distance threshold and number of selected variables in Physionet with Fixed Distance Method.

DELAY	DIMENSION	1	3	4	5	6	7	8	9
1		0.8920	**0.9012**	0.8995	0.8985	0.8979	0.8997	0.8998	0.8972
		82.49%	**83.19**%	83.28%	83.32%	83.11%	83.18%	82.84%	83.24%
		1–9 VAR	**1.2–10 VAR**	1.2–9 VAR	1.4–7 VAR	1.6–6 VAR	1.6–6 VAR	1.6–10 VAR	1.8–7 VAR
2			0.8938	0.8953	0.8970	0.8972	0.8979	0.8997	0.8982
			82.31%	82.48%	82.49%	82.40%	82.52%	82.71%	82.61%
			0.8–13 VAR	1–15 VAR	1–15 VAR	1.2–11 VAR	1.4–10 VAR	1.6–11 VAR	1.8–12 VAR
3			0.8880	0.8946	0.8964	0.8955	0.8926	0.8913	0.8891
			82.11%	82.15%	82.40%	82.44%	81.83%	81.76%	81.93%
			1–14 VAR	0.8–11 VAR	1.2–15 VAR	1.4–13 VAR	1.4–13 VAR	1.6–13 VAR	2–15 VAR
4			0.8903	0.8914	0.8995	0.8979	0.8941	0.8901	0.8875
			83.14%	82.84%	83.36%	83.17%	82.89%	82.49%	81.84%
			1.2–11 VAR	1.2–13 VAR	1.2–8 VAR	1.4–13 VAR	1.6–10 VAR	1.8–10VAR	2–11 VAR
5			0.8852	0.8924	0.8930	0.8932	0.8904	0.8883	0.8862
			83.09%	82.91%	83.28%	82.75%	82.49%	82.24%	82.09%
			1–11 VAR	1–16 VAR	1.2–12 VAR	1.4–13 VAR	1.8–15 VAR	1.8–12 VAR	2–13 VAR
6			0.8832	0.8803	0.8932	0.8897	0.8933	0.8873	0.8866
			82.27%	81.15%	82.33%	81.95%	82.54%	82.40%	82.09%
			1.2–12 VAR	1.2–13 VAR	1.2–14 VAR	1.4–11 VAR	1.6–13 VAR	2–12 VAR	2.2–14 VAR
7			0.8838	0.8930	0.8964	0.8972	0.8950	0.8906	0.8895
			82.48%	82.22%	82.63%	82.95%	82.85%	82.48%	82.33%
			1–12 VAR	1–13 VAR	1.2–14 VAR	1.4–14 VAR	1.6–15 VAR	2–15 VAR	2–16 VAR
8			0.8813	0.8891	0.8902	0.8929	0.8897	0.8863	0.8826
			82.44%	82.12%	82.22%	82.85%	82.23%	82.33%	81.64%
			1–17 VAR	1–12 VAR	1.2–12 VAR	1.4–16 VAR	1.6–13 VAR	2–15 VAR	2.2–14 VAR
9			0.8827	0.8892	0.8943	0.8915	0.8883	0.8847	0.8796
			82.23%	82.50%	83.11%	82.47%	82.33%	81.98%	81.32%
			1–16 VAR	1–11 VAR	1.2–11 VAR	1.6–15 VAR	1.8–16 VAR	1.8–14 VAR	2.2–15 VAR
10			0.8811	0.8918	0.8946	0.8914	0.8871	0.8818	0.8763
			82.12%	82.49%	82.69%	82.46%	82.04%	81.77%	81.07%
			1–16 VAR	1–12 VAR	1.2–11 VAR	1.4–12 VAR	1.6–14 VAR	2–12 VAR	2.2–11 VAR
12			0.8813	0.8924	0.8907	0.8827	0.8798	0.8769	0.8719
			81.88%	82.37%	82.11%	81.82%	81.27%	81.06%	80.80%
			0.8–16 VAR	1–14 VAR	1.2–13 VAR	1.6–13 VAR	1.8–16 VAR	2–12 VAR	2.2–13 VAR

**Table 3:** AUCs, accuracies, FAN and number of selected variables in Physionet with FAN method.

DELAY	DIMENSION	1	3	4	5	6	7	8	9
1		0.8988	0.9074	0.9058	0.9143	0.9116	0.9055	0.9070	0.9088
		83.22%	83.50%	83.52%	84.48%	84.70%	83.91%	84.16%	84.12%
		20%–16 VAR	15%–15 VAR	20%–12 VAR	20%–15 VAR	20%–14 VAR	10%–15 VAR	7.5%–11 VAR	5%–15 VAR
2			0.9060	0.9061	0.9015	0.9076	0.9099	0.9130	0.9159
			84.25%	84.44%	83.11%	84.88%	84.39%	84.41%	84.63%
			17.5%–15 VAR	20%–12 VAR	5%–9 VAR	20%–12 VAR	5%–15 VAR	5%–12 VAR	5%–12 VAR
3			0.9048	0.9090	0.9136	0.9166	0.9187	0.9207	0.9191
			84.32%	84.99%	85.33%	85.62%	85.88%	86.33%	85.50%
			17.5%–13 VAR	5%–10 VAR	5%–12 VAR	7.5%–12 VAR	5%–13 VAR	5%–11 VAR	7.5%–11 VAR
4			0.9099	0.9178	0.9208	0.9215	**0.9253**	0.9217	0.9198
			85.12%	85.68%	86.23%	86.22%	**85.76%**	85.73%	84.83%
			7.5%–8 VAR	10%–8 VAR	7.5%–9 VAR	10%–8 VAR	**5%–9 VAR**	7.5%–9 VAR	5%–7VAR
5			0.9167	0.9212	0.9227	0.9213	0.9209	0.9205	0.9184
			85.30%	86.19%	86.30%	85.96%	85.73%	85.72%	85.38%
			5%–10 VAR	5%–9 VAR	5%–9 VAR	5%–7 VAR	5%–9 VAR	5%–9 VAR	5%–10 VAR
6			0.9170	0.9188	0.9183	0.9182	0.9186	0.9167	0.9148
			85.02%	85.31%	85.27%	85.19%	85.33%	85.21%	84.98%
			5%–10 VAR	5%–7 VAR	5%–8 VAR	5%–9 VAR	5%–9 VAR	5%–10 VAR	5%–9 VAR
7			0.9172	0.9213	0.9221	0.9211	0.9182	0.9160	0.9136
			85.42%	85.68%	85.65%	85.54%	85.05%	85.03%	84.69%
			7.5%–12 VAR	5%–7 VAR	5%–9 VAR	5%–9 VAR	5%–9 VAR	5%–9 VAR	5%–8 VAR
8			0.9156	0.9190	0.9191	0.9180	0.9154	0.9129	0.9098
			85.30%	85.53%	85.63%	85.47%	85.11%	84.80%	84.16%
			7.5%–12 VAR	5%–7 VAR	5%–8 VAR	5%–8 VAR	5%–8 VAR	5%–9 VAR	5%–8 VAR
9			0.9128	0.9158	0.9170	0.9142	0.9125	0.9079	0.9046
			85.15%	85.32%	85.52%	85.08%	84.64%	84.62%	84.63%
			7.5%–12VAR	5%–7 VAR	5%–7 VAR	5%–7 VAR	5%–8 VAR	5%–7 VAR	7.5%–9 VAR
10			0.9115	0.9144	0.9135	0.9119	0.9086	0.9051	0.9020
			84.96%	84.85%	84.79%	84.67%	84.35%	84.49%	84.07%
			7.5%–7 VAR	5%–7 VAR	5%–7 VAR	5%–7 VAR	5%–7 VAR	7.5%–9 VAR	7.5%–9 VAR
12			0.9052	0.9065	0.9063	0.9039	0.9010	0.8993	0.8969
			83.87%	84.39%	84.31%	84.27%	84.12%	83.53%	83.43%
			7.5%–7 VAR	5%–7 VAR	5%–10 VAR	17.5%–9 VAR	15%–13 VAR	15%–13 VAR	17.5%–12 VAR

**Table 4:** AUCs, accuracies, distance threshold and number of selected variables in HuGCDN2014 with Fixed Distance Method.

DELAY	DIMENSION	1	3	4	5	6	7	8	9
1		0.8004	0.7973	0.7702	0.7775	0.7533	0.7560	0.7518	0.7542
		79.30%	78.99%	78.19%	78.58%	78.52%	78.96%	78.22%	78.46%
		2–10 VAR	2–11 VAR	1.8–5 VAR	2.2–9 VAR	1.2–7 VAR	1.2–8 VAR	1.4–10 VAR	1.6–7 VAR
2			0.7812	0.7780	0.7762	0.7825	0.7857	0.7929	0.7854
			77.80%	79.53%	78.44%	78.44%	78.77%	78.60%	79.23%
			1.2–12 VAR	1–11 VAR	1.4–5 VAR	1.6–5 VAR	1.8–5 VAR	2–6 VAR	1.8–5 VAR
3			0.7693	0.7767	0.7724	0.7782	0.7843	0.8029	0.8024
			77.46%	78.42%	79.15%	77.33%	77.70%	79.95%	78.86%
			1.4–6 VAR	1.6–5 VAR	1.4–5 VAR	1.6–8 VAR	1.8–8 VAR	1.8–8 VAR	2.2–7 VAR
4			0.7772	0.7714	0.7813	0.7967	0.8079	0.8142	0.8184
			78.22%	78.13%	78.18%	79.81%	79.66%	79.86%	80.69%
			1.4–7 VAR	1.4–7 VAR	1.4–8 VAR	1.4–6 VAR	1.8–6 VAR	2–8 VAR	2–7 VAR
5			0.7796	0.7980	0.8068	0.8092	0.8078	0.8231	0.8253
			78.29%	79.95%	79.17%	79.98%	79.70%	80.76%	80.71%
			1.4–6 VAR	1–9 VAR	1.4–9 VAR	1.6–11 VAR	1.8–7 VAR	2–11 VAR	2.2–8 VAR
6			0.7673	0.7759	0.7910	0.8163	0.8256	0.8243	0.8225
			78.95%	76.58%	78.63%	79.71%	80.82%	80.75%	80.57%
			1.8–9 VAR	0.61–12 VAR	1.4–8 VAR	1.6–9 VAR	1.8–11 VAR	2–9 VAR	2.2–8 VAR
7			0.7678	0.7938	0.8033	0.8163	0.8277	0.8259	0.8238
			78.96%	77.37%	80.15%	80.39%	80.88%	80.33%	80.45%
			1.4–9 VAR	0.61–12 VAR	1.4–9 VAR	1.6–7 VAR	1.8–11 VAR	2–8 VAR	2.2–8 VAR
8			0.8139	0.8257	0.8286	**0.8326**	0.8311	0.8284	0.8275
			79.93%	81.01%	80.04%	**81.73%**	82.00%	81.15%	80.34%
			0.8–9 VAR	1–13 VAR	1.4–10 VAR	**1.6–12 VAR**	1.8–11 VAR	2–8 VAR	2.2–8 VAR
9			0.8028	0.8245	0.8301	0.8315	0.8297	0.8294	0.8282
			79.94%	80.87%	80.51%	81.02%	80.28%	80.78%	79.84%
			0.8–7 VAR	1–13 VAR	1.4–12 VAR	1.6–12 VAR	1.8–12 VAR	2–12 VAR	2.2–11 VAR
10			0.7807	0.8188	0.8239	0.8233	0.8211	0.8205	0.8205
			79.41%	80.87%	80.54%	80.24%	81.02%	80.68%	80.18%
			2.2–12 VAR	1–12 VAR	1.4–12 VAR	1.6–12 VAR	1.8–10 VAR	2–11 VAR	2.2–11 VAR
12			0.7876	0.7977	0.8087	0.8083	0.8061	0.8155	0.8008
			80.30%	79.31%	79.78%	79.67%	79.77%	79.47%	78.81%
			2.2–11 VAR	1.2–10 VAR	1.2–11 VAR	1.4–10 VAR	1.6–7 VAR	1.8–11 VAR	2.2–6 VAR

**Table 5:** AUCs, accuracies, FAN and number of selected variables in HuGCDN2014 with FAN method.

DELAY	DIMENSION	1	3	4	5	6	7	8	9
1		0.7964	0.8248	0.8151	0.8128	0.8150	0.8226	0.8227	0.8188
		80.89%	80.79%	79.80%	80.30%	80.48%	80.90%	80.78%	80.69%
		15%–7 VAR	20%–11 VAR	17.5%–13 VAR	20%–10 VAR	20%–10 VAR	20%–10 VAR	17.5%–10 VAR	15%–10 VAR
2			0.8192	0.8217	0.8268	0.8345	0.8382	0.8437	0.8445
			80.88%	81.59%	81.30%	81.27%	81.02%	81.33%	82.30%
			15%–13 VAR	2.5%–10 VAR	2.5%–11 VAR	20%–11 VAR	20%–11 VAR	20%–12 VAR	12.5%–14 VAR
3			0.8240	0.8343	0.8353	0.8410	0.8449	0.8476	0.8486
			81.09%	82.18%	82.04%	81.01%	80.93%	81.68%	82.11%
			7.5%–11 VAR	5%–11 VAR	5%–10 VAR	7.5%–13 VAR	5%–9 VAR	10%–10 VAR	10%–10 VAR
4			0.8358	0.8417	0.8464	0.8407	0.8458	0.8541	0.8542
			82.66%	82.81%	83.15%	82.09%	82.25%	82.49%	83.44%
			5%–10 VAR	2.5%–5 VAR	2.5%–7 VAR	10%–15 VAR	12.5%–11 VAR	2.5%–8 VAR	5%–10 VAR
5			0.8443	0.8491	0.8520	0.8591	0.8585	**0.8622**	0.8581
			82.59%	83.23%	83.12%	83.23%	83.61%	**83.64%**	84.18%
			2.5%–5 VAR	5%–7 VAR	5%–9 VAR	5%–9 VAR	5%–8 VAR	**5%–10 VAR**	5%–6 VAR
6			0.8429	0.8478	0.8540	0.8571	0.8588	0.8574	0.8599
			82.95%	83.02%	83.37%	83.49%	83.46%	83.57%	83.70%
			5%–7 VAR	5%–8 VAR	5%–8 VAR	5%–6 VAR	5%–7 VAR	5%–10 VAR	5%–6 VAR
7			0.8439	0.8485	0.85644	0.8578	0.8528	0.8511	0.8495
			83.28%	83.28%	83.49%	83.56%	83.73%	83.52%	83.52%
			5%–7 VAR	5%–9 VAR	5%–7 VAR	5%–6 VAR	5%–10 VAR	5%–8 VAR	5%–10 VAR
8			0.8494	0.8515	0.85638	0.8526	0.8526	0.8521	0.8522
			82.94%	83.33%	83.64%	83.76%	83.67%	83.69%	83.63%
			5%–6 VAR	5%–7 VAR	5%–6 VAR	5%–10 VAR	7.5%–9 VAR	7.5%–9 VAR	7.5%–8 VAR
9			0.8503	0.8538	0.8518	0.8517	0.8518	0.8531	0.8536
			83.06%	83.65%	83.40%	83.57%	83.76%	83.03%	84.04%
			5%–6 VAR	5%–6 VAR	5%–7 VAR	5%–9 VAR	10%–8 VAR	7.5%–8 VAR	10%–7 VAR
10			0.8467	0.8500	0.8534	0.8520	0.8523	0.8528	0.8492
			82.69%	82.91%	83.58%	83.17%	83.09%	83.09%	82.59%
			5%–7 VAR	7.5%–8 VAR	5%–6 VAR	5%–8 VAR	7.5%–7 VAR	7.5%–7 VAR	7.5%–6 VAR
12			0.8450	0.8449	0.8474	0.8471	0.8488	0.8518	0.8508
			81.63%	82.57%	83.26%	81.80%	83.08%	83.45%	83.10%
			10%–9 VAR	20%–12 VAR	15%–8 VAR	17.5%–8 VAR	10%–8 VAR	10%–7 VAR	10%–9 VAR

**Fig 3:**
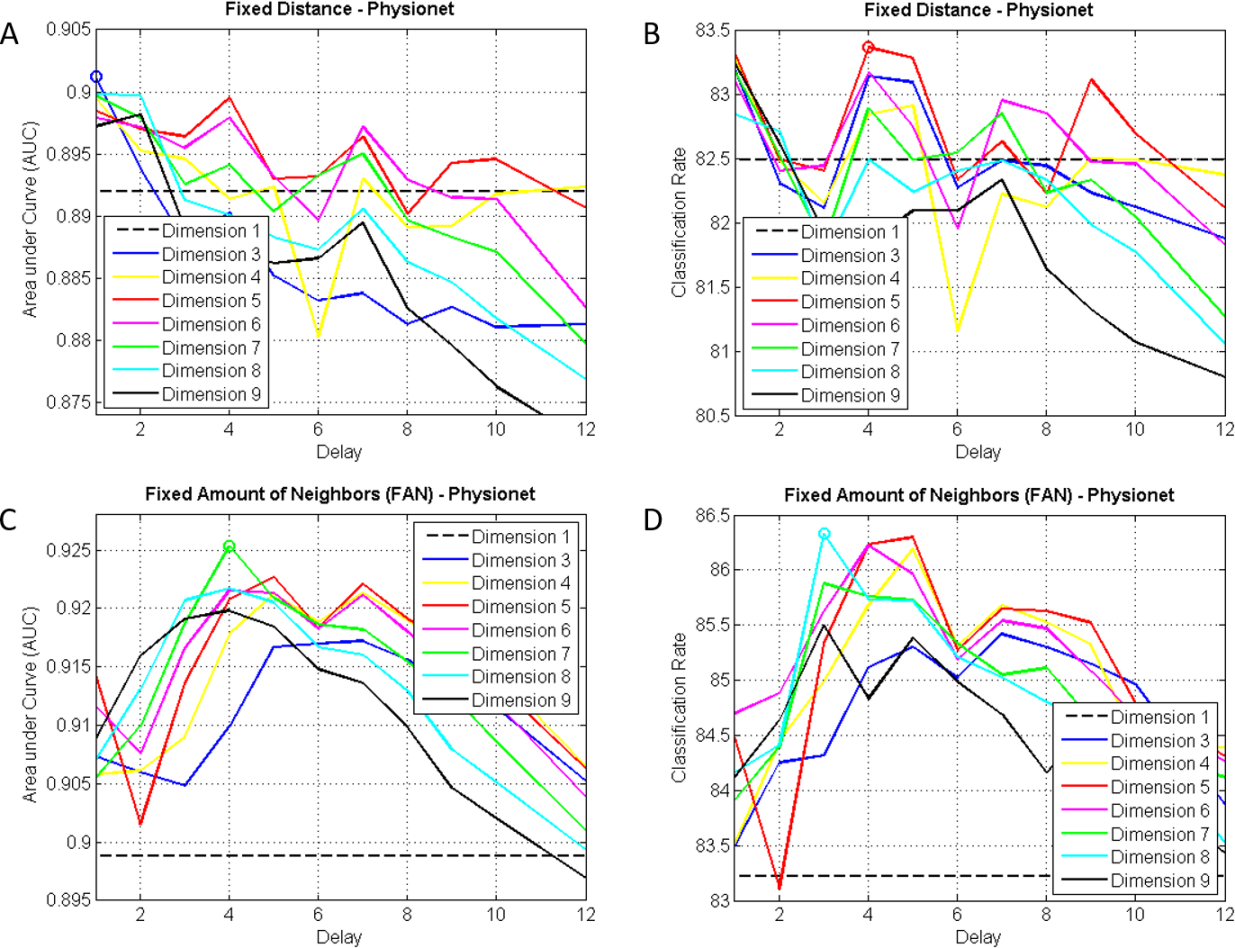
AUCs and accuracies for different dimensions and delays in Physionet. (A and B) Fixed Distance Method. (C and D) FAN Method.

**Fig 4:**
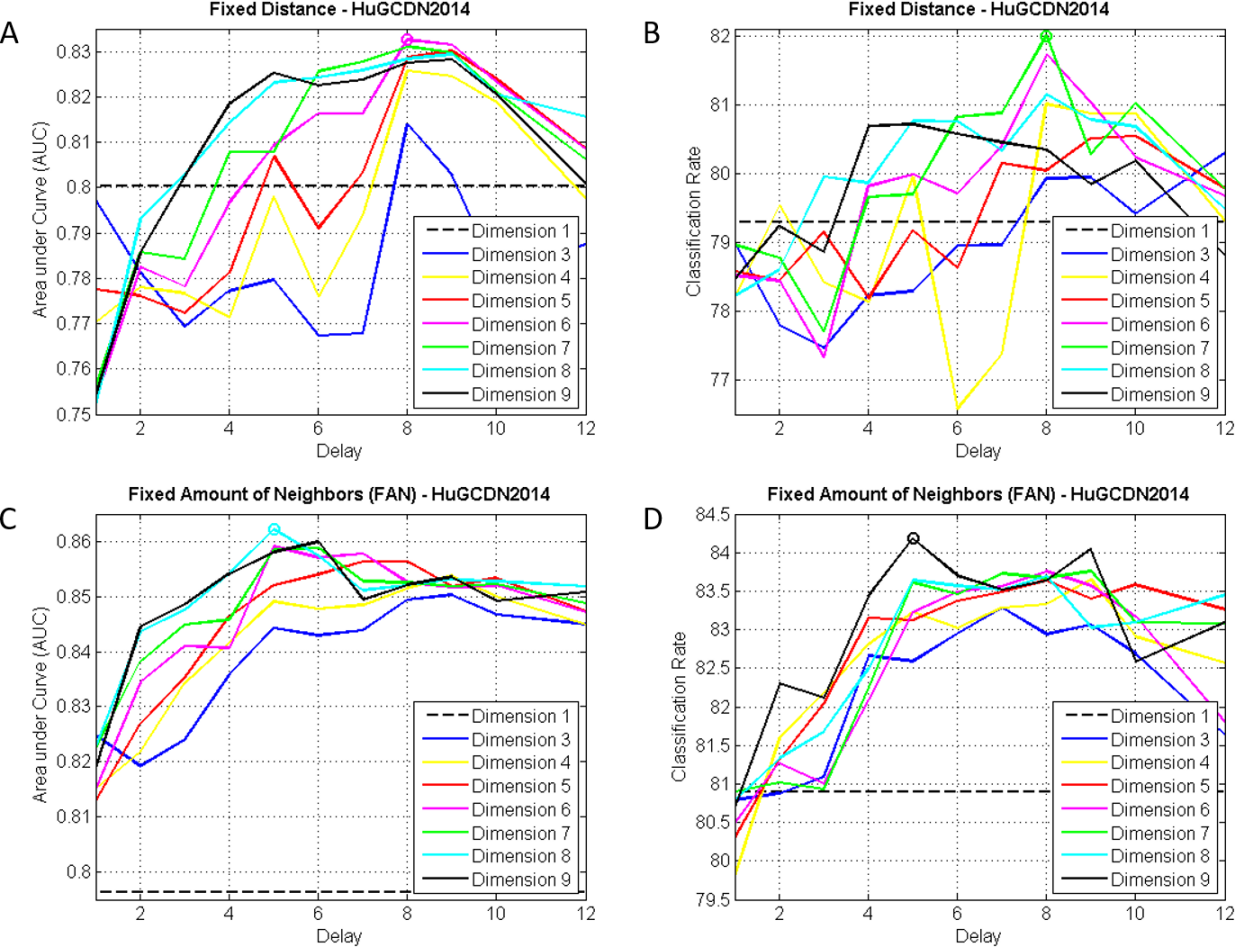
AUCs and accuracies for different dimensions and delays in HuGCDN. (A and B) Fixed Distance Method. (C and D) FAN Method.

Figs 3 and 4 show how AUCs and accuracies evolve depending on the dimension and the delay for both databases. The possibility of non embedding is represented by dimension 1. The best results in Physionet are AUC = 0.93 (dimension = 7, delay = 4, FAN-5%) and Acc = 86.33% (dimension = 8, delay = 3, FAN-5%), and in HuGCDN2014, AUC = 0.86 (dimension = 8, delay = 5, FAN-5%) and Acc = 84.18% (dimension = 9, delay = 5, FAN-5%).

In Tables 2 and 4, representing the results for the Fixed Distance Method, we can see a delay that is best for every dimension. For Physionet, delay 1 is the best, and for HuGCDN2014 delays around 8–9 yield the best results. In Table 2, for dimensions 5 and 9, delay 1 was chosen, as AUC values were very similar to the best ones and included fewer variables. As far as the distance threshold is concerned, we can see that, in the best cases (Tables 2 and 4), increasing the dimension implies that higher values are needed. In Physionet, values increase from 1.2 to 1.8, and in HuGCDN2014 from 0.8 to 2.2. This might imply that increasing the dimension also increases the distance between the points of the phase space. Therefore, a higher distance is needed to include enough neighbours in the neighbourhood, thus allowing us to extract the recurrence information.

However, the results obtained using the FAN Method, shown in Tables 3 and 5, differ significantly from the previous ones. In general, better AUCs and accuracies are reached. In this case, the outcomes for both databases are rather similar. To achieve the best results, a lower delay is needed when increasing the dimension. Moreover, there is a very interesting finding we consider a novel contribution in the RQA analysis. For dimensions over 5, there is a tendency towards stabilization in the optimal delay value (see Tables 3 and 5). As this behaviour is independent of the database, we would expect the same evolution in other datasets. The best results are reached in both databases for delays around 4–5, dimensions around 7–8, and 5% of neighbours is preferred in most cases. Only in a few cases do 7.5% or 10% yield better results. As in the FAN Method this percentage represents the density of recurrence points in each of the columns, we can see that the best value coincides with the REC proposed by other authors [41].

### Feature selection

The RQA measures of HRV commonly used for sleep apnea detection are: REC, DET, L, Lmax, ENTR, LAM, TT, Vmax. T1, T2, RPDE and Trans were first used in this context by Le et al. [11]. However, no author has used other features, like the Clustering Coefficient (Clust), Mean Recurrence Time (RT), maximal Recurrence Time (RTmax), Recurrence Frequency (RF) and Entropy of the White Vertical Lines (ENTRW), before in sleep apnea. Therefore, we introduce them in our experiments to evaluate the system and to uncover the importance of these features in the system performance and, by extension, in the characterization of sleep apnea from a cardiac rate point of view.

**Fig 5:**
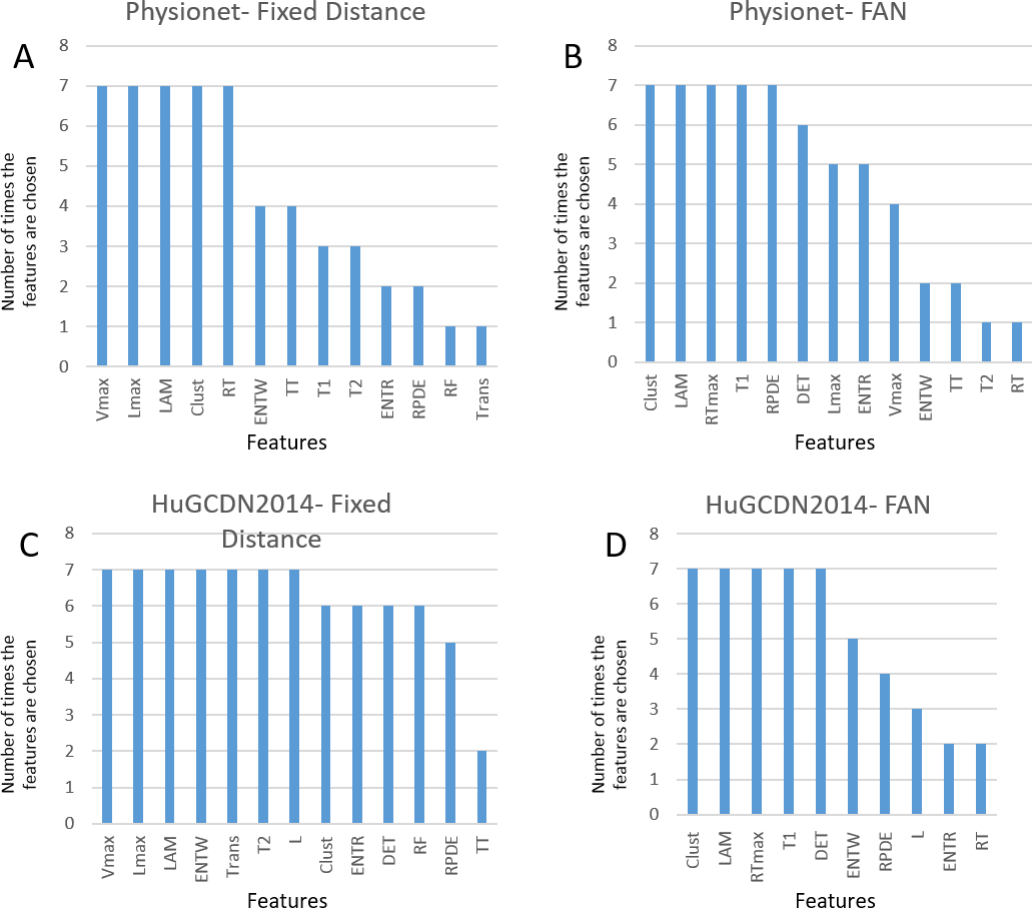
**Number of times the features are chosen for the best combinations dimension (*m*)/delay(***τ***) in:** (A) Physionet-Fixed Distance, (B) Physionet-FAN, (C) HuGCDN2014-Fixed Distance and (D) HuGCDN2014-FAN.

Tables 6–9 show the rankings of selected features for each case: Physionet database using the Fixed Distance Method, Physionet database using the FAN Method, and in the same way for the HuGCDN2014 database. The first column contains the best combinations dimension (*m*)-delay(*τ*), columns 3 and 4 show the AUCs and accuracies, respectively, and the last column, the number of selected features. In Tables 6 and 8 (Fixed Distance Method), the second column represents the best values to multiply by the frame standard deviation (*σ*). In Tables 7 and 9 (FAN Method) the second column shows the percentage of neighbours to be considered. The selected features appear in the same order as in the ranking described in section 2.5. In all four tables, the cells containing features repeated in each of the combinations are highlighted in grey. Fig 5 is a summary of the previous tables. There we find the number of times the features are chosen for the best combinations dimension (*m*)-delay(*τ*).

**Table 6:** Features selected in Physionet (Fixed Distance Method) in the best combinations dimension(*m*)/delay(*τ*).

*m-τ*	σ	AUC	Acc(%)	Selected Features										Var
3–1	1.2	0.9012	83.19	Vmax	Lmax	ENTR	LAM	T2	RT	Clust	Trans	TT	RPDE	10
4–1	1.2	0.8995	83.28	Vmax	Lmax	RT	T2	LAM	TT	Clust	ENTR	T1		9
5–1	1.4	0.8985	83.32	Vmax	Lmax	RT	LAM	Clust	TT	T2				7
6–1	1.6	0.8979	83.11	Vmax	Lmax	LAM	RT	Clust	ENTW					6
7–1	1.6	0.8997	83.18	Vmax	Lmax	RT	LAM	ENTW	Clust					6
8–1	1.6	0.8998	82.84	Vmax	RT	Lmax	ENTW	Clust	LAM	RPDE	RF	T1	TT	10
9–1	1.8	0.8972	83.24	Vmax	RT	Lmax	Clust	ENTW	LAM	T1				7

**Table 7:** Features selected in Physionet (FAN Method) in the best combinations dimension(*m*)/delay(*τ*).

*m-τ*	FAN(%)	AUC	Acc(%)	Selected Features												Var
3–7	7.5	0.9172	85.42	T1	LAM	Clust	RTmax	DET	RT	TT	Vmax	ENTR	T2	RPDE	Lmax	12
4–7	5	0.9213	85.68	T1	LAM	RTmax	Clust	DET	RPDE	ENTR						7
5–5	5	0.9227	86.30	T1	LAM	RTmax	DET	ENTR	Clust	RPDE	Lmax	ENTW				9
6–4	10	0.9215	86.22	T1	Clust	LAM	Vmax	RTmax	Lmax	TT	RPDE					8
7–4	5	0.9253	85.76	Clust	LAM	RTmax	T1	RPDE	Lmax	DET	ENTR	ENTW				9
8–4	7.5	0.9217	85.73	T1	Clust	LAM	RTmax	Vmax	DET	RPDE	Lmax	ENTR				9
9–4	5	0.9198	84.83	Clust	LAM	T1	RTmax	DET	RPDE	Vmax						7

**Table 8:** Features selected in HuGCDN2014 (Fixed Distance Method) in the best combinations dimension(*m*)/delay(*τ*).

*m-τ*	σ	AUC	Acc(%)	Selected Features													Var
3–8	0.8	0.8139	79.93	ENTR	Vmax	ENTW	Lmax	Trans	L	LAM	TT	T2					9
4–8	1	0.8257	81.01	ENTW	Vmax	T2	ENTR	LAM	Lmax	L	DET	Trans	TT	Clust	RF	RPDE	13
5–9	1.4	0.8301	80.51	ENTR	Vmax	Trans	Lmax	LAM	Clust	DET	ENTW	L	T2	RPDE	RF		12
6–8	1.6	0.8326	81.73	LAM	Lmax	ENTW	Vmax	Trans	Clust	T2	DET	RPDE	L	ENTR	RF		12
7–8	1.8	0.8311	82.00	LAM	Clust	Lmax	ENTW	Vmax	T2	DET	Trans	L	RPDE	RF			11
8–9	2	0.8294	80.78	LAM	Vmax	Clust	Lmax	T2	ENTW	DET	Trans	ENTR	RF	RPDE	L		12
9–9	2.2	0.8282	79.84	LAM	Vmax	Clust	Lmax	ENTW	DET	T2	ENTR	Trans	RF	L			11

**Table 9:** Features selected in HuGCDN2014 (FAN Method) in the best combinations dimension(*m*)/delay(*τ*).

*m-τ*	FAN(%)	AUC	Acc(%)	Selected Features										Var
3–9	5	0.8503	83.06	LAM	T1	RTmax	Clust	DET	ENTW					6
4–9	5	0.8538	83.65	LAM	T1	DET	RTmax	Clust	RPDE					6
5–7	5	0.8564	83.49	LAM	DET	T1	ENTW	RTmax	Clust	RPDE				7
6–5	5	0.8591	83.23	DET	LAM	Clust	ENTW	RTmax	L	T1	RT	ENTR		9
7–6	5	0.8588	83.46	LAM	T1	DET	ENTW	L	RTmax	Clust				7
8–5	5	0.8622	83.64	LAM	DET	Clust	L	T1	RTmax	ENTW	RT	RPDE	ENTR	10
9–6	5	0.8599	83.70	LAM	T1	DET	RTmax	Clust	RPDE					6

**Fig 6:**
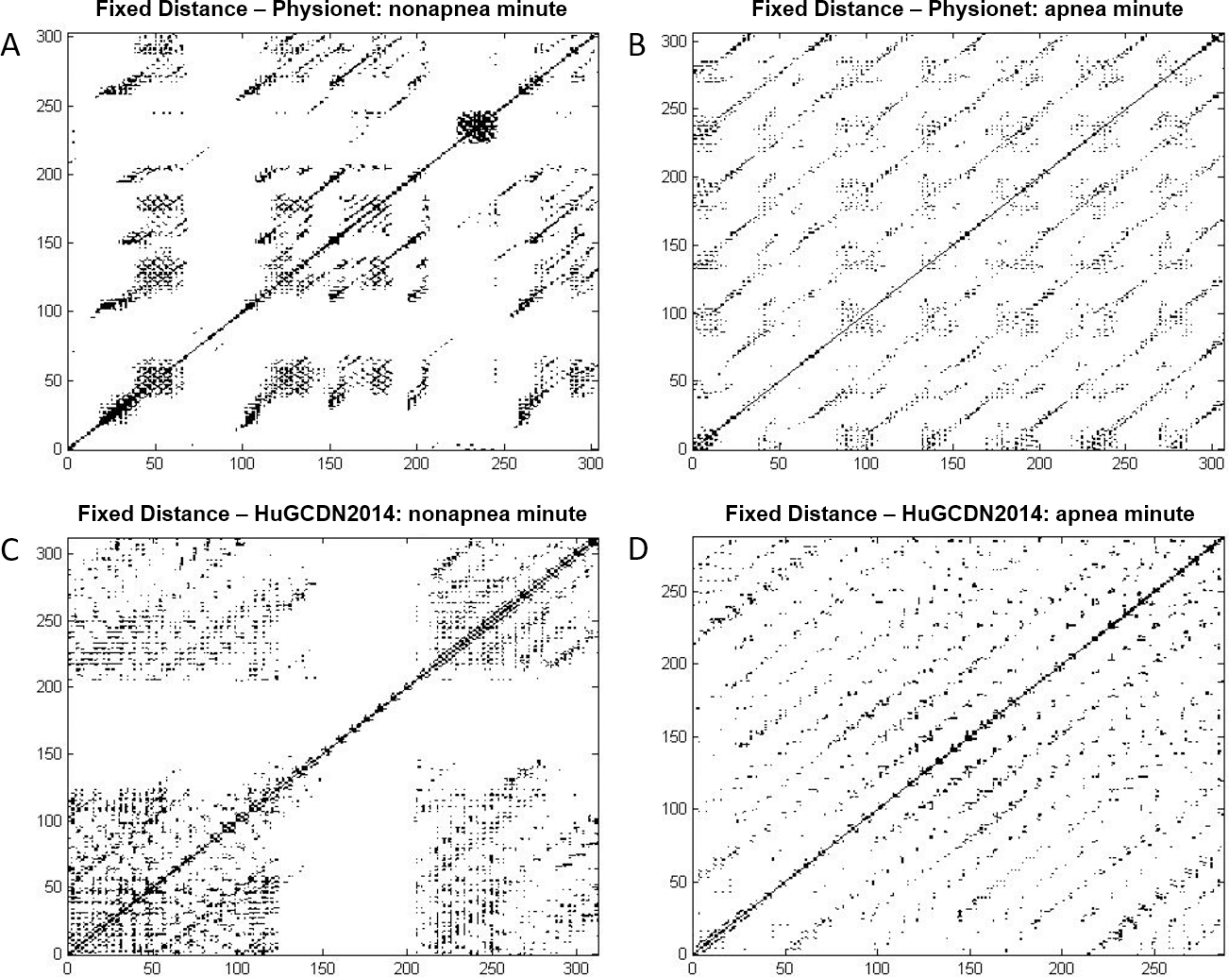
Recurrence plots with Fixed Distance Method. (A and B) Physionet: *m* = 5, *τ* = 3, 1.2σ. (C and D) HuGCDN2014: m = 7, τ = 8, 1.8σ.

**Fig 7:**
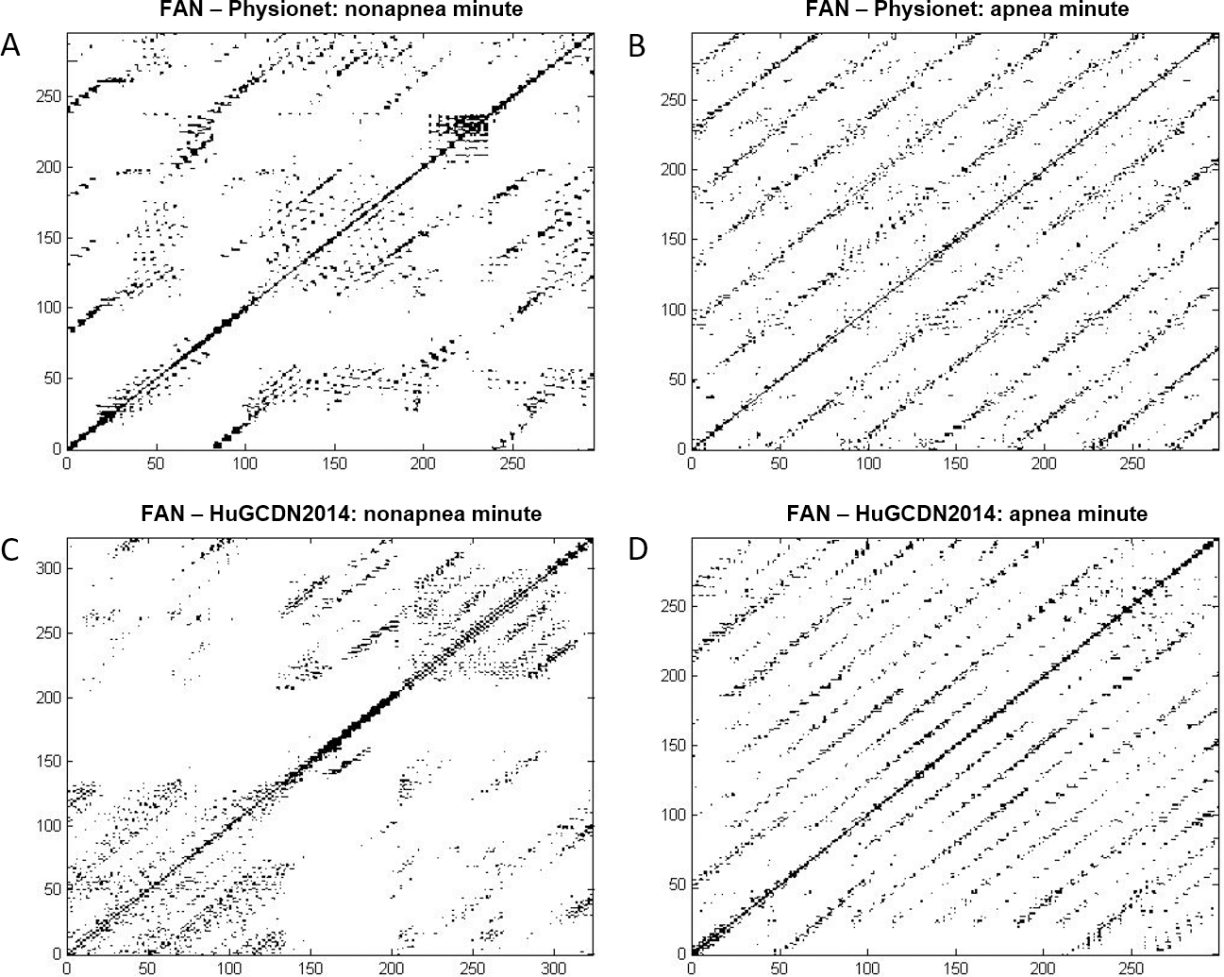
Recurrence plots with FAN method. (A and B) Physionet: m = 5, *τ* = 5, FAN: 5%. (C and D) HuGCDN2014: m 7, τ = 6, FAN: 5%.

Analyzing Fig 5, we can see that the selected features are different, depending on the method chosen to define the distance threshold. In the Fixed Distance Method (see Tables 6 and 8), there are three variables that play an especially important role discriminating apnea and nonapnea minutes: Vmax, Lmax and LAM. Vmax always comes first in the ranking in Physionet. However, in the HuGCDN2014, although Vmax is among the top positions, LAM comes first. From these results, we can see that the vertical structures (Vmax and LAM) in RPs are in first place, and diagonal structures (Lmax) in second place, for classification purposes. The latter can be justified because diagonal structures are also found in the RPs of nonapnea minutes (Figs 6 and 7), due to de RSA component, although they are not as notable as in the apnea minutes, where the CVHR is predominant. Furthermore, there are two variables that play an important role for apnea minute quantification, and are considered after the feature selection process in both databases. They belong to the group of features not used before in this context: Clust and ENTW. The remaining selected features seem to be database dependent. In Physionet, the RT, included in the subset of newly used features, is chosen for every dimension, and T1, TT and T2 are also considered in 3–4 cases out of 7. The remaining variables are occasionally selected: ENTR, RPDE, RF and Trans, depending on the dimension. However, in the HuGCDN2014 database, results are more stable. Practically the same set of features is selected for all dimensions, except for TT, that is only chosen twice. The other selected features, listed according to their position in the ranking are: ENTR, T2, Trans, L, DET, RPDE and RF.

**Fig 8:**
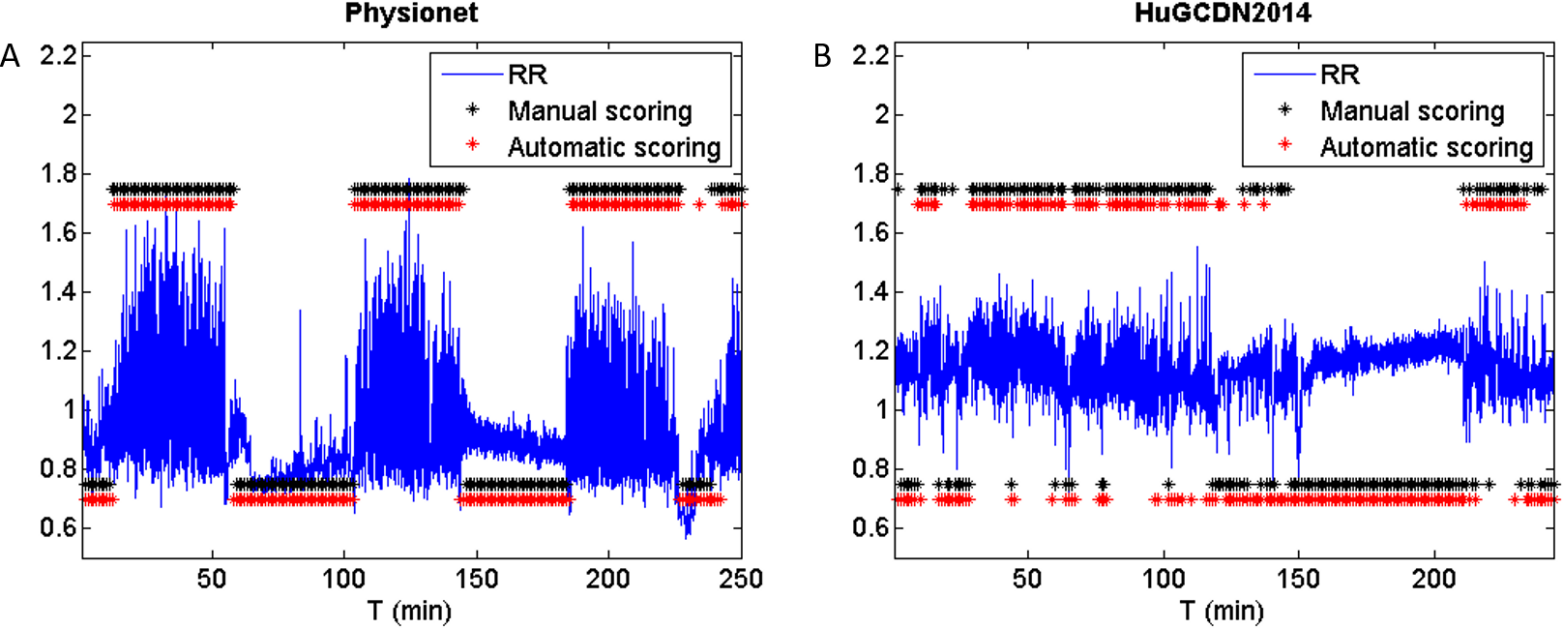
RR series of an OSA-diagnosed patient, per-segment manual scoring and per-segment automatic scoring (high level = apnea; low level = nonapnea). (A) Physionet: *m* = 7, *τ* = 4, FAN: 0.05%. (B) HuGCDN2014: m = 8, τ = 5, FAN:0.05%.

In the FAN Method, we find for both databases five variables with an outstanding role in the classification process: Clust, LAM, RTmax, T1 and DET. Clust, originating in the complex network theory, RTmax and T1, related to recurrence times, LAM, a vertical characteristic, and DET, a diagonal one. According to Tables 7 and 9, DET is the preferred feature from the diagonal ones, instead of Lmax. But, here, as in the Fixed Distance Method, the vertical feature LAM stands out against the diagonal variable, DET. Especially interesting is the inclusion of Clust and RTmax because neither is commonly used in the studies where RQA is applied to HRV in sleep apnea. Comparing our outcomes with those obtained by Nguyen et al. [13], the only authors that have introduced the FAN method for sleep apnea quantification, we can see that DET, LAM and T1 are also included in the set of selected features. In particular, DET and LAM are always chosen regardless of the percentage of neighbours considered, reinforcing again the importance of diagonal and vertical measures in RPs. Fig 8 shows, for both databases, RR series of an OSA-diagnosed patient, the per-segment manual scoring performed by the practitioner, and the per-segment automatic scoring obtained by the selected parameters. Fig 8A and 8B are obtained according to the best performing parameter values (see Tables 7 and 9).

In summary, we can see that the newly introduced features for sleep apnea quantification play an important role in the classification process. These variables enrich the feature vector and improve both AUCs and accuracies, compared to the results obtained with the most commonly used RQA measures. This implies that there is additional information in the RPs that can be extracted to capture the intergroup differences. Especially important are the clustering coefficient and two features related to the white vertical lines: the maximal Recurrence Time (RTmax) and the Entropy of the White Vertical Lines (ENTRW). Furthermore, features related to the white vertical lines are linked to recurrence time information. This strengthens the relevance of these variables in the characterization of the physiological process. In fact, Webber et al. [63] already pointed out the importance of recurrence times to quantify the periodicities present in dynamical systems as they are able to reveal subtle characteristics of physiological signals. However, results also show there are some features that provide less useful information for classification purposes. Hence, they appear not to be very closely related to the cardiorespiratory dynamic, as they are almost always ruled out in the feature selection process. In particular, REC, TT and Trans.

**Fig 9:**
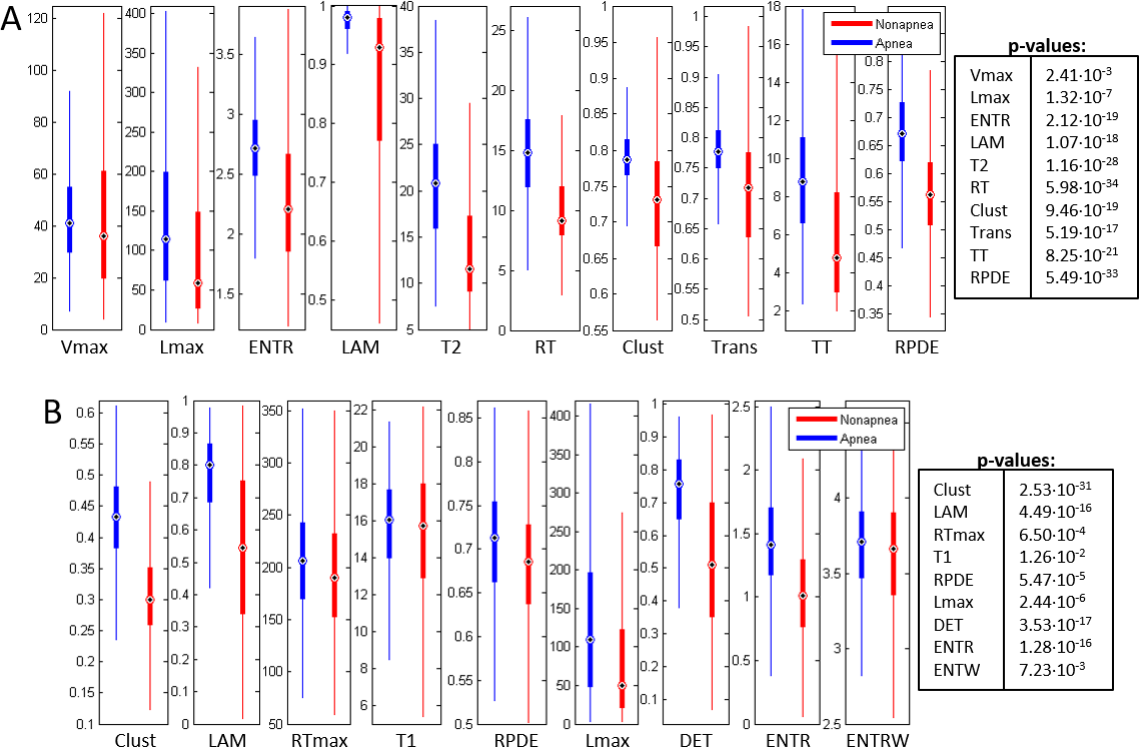
Boxplots of the selected features in Physionet for the best combination dimension (*m*)/delay(*τ*). (A) Fixed Distance Method. (B) FAN method.

**Fig 10:**
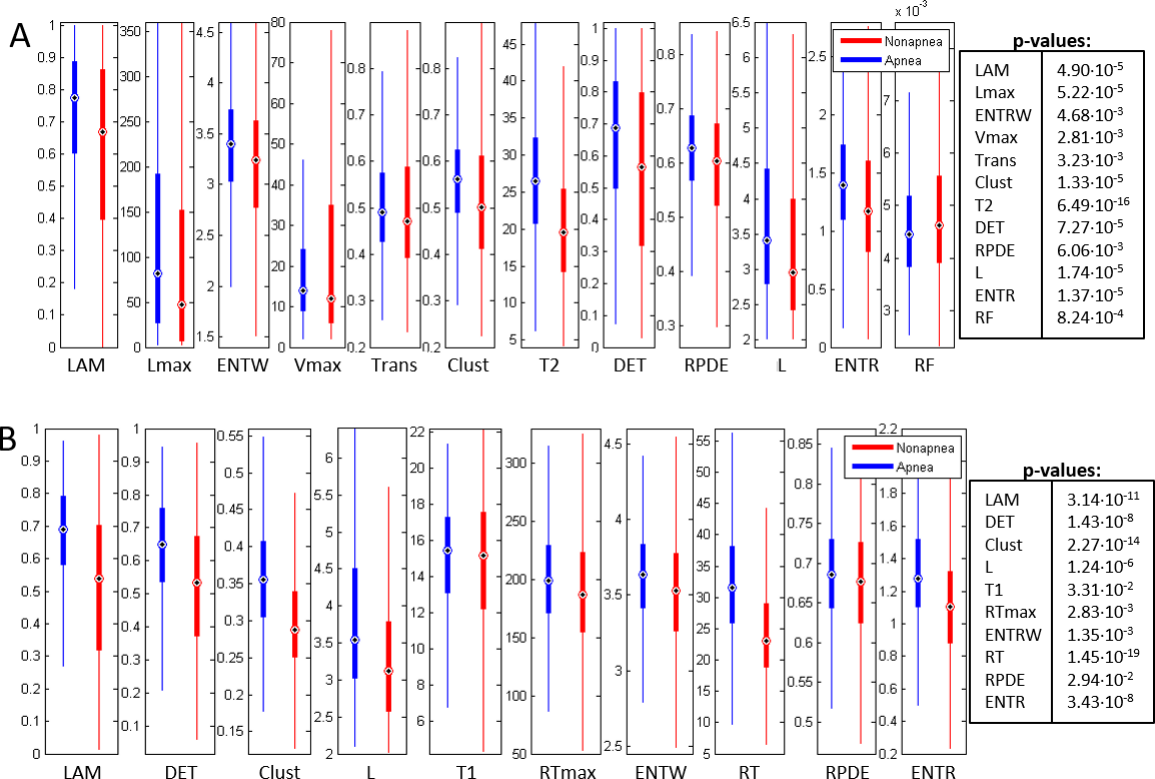
Boxplots of the selected features in HuGCDN2014 for the best combination dimension (*m*)/delay(*τ*). (A) Fixed Distance Method. (B) FAN Method.

The values and observed differences in the RQA measures between apnea and nonapnea minutes were visualized as box-plots representing the median, the first and the third quartile (see Figs 9 and 10). Only the selected features for the best combinations dimension (*m*)/delay(*τ*) in Physionet (Fixed Distance and FAN) and HuGCDN2014 (Fixed Distance and FAN) are included.

## Discussion

This is a novel study that performs a thorough exploratory analysis for sleep apnea detection using RQA applied to HRV, in which the contributions to the state of the art are twofold. On the one hand, we focus on finding reference values for the different parameters implied in the RQA approach, namely dimension, delay and distance threshold, when HRV is analyzed in the context of sleep apnea as, until now, there is no agreement about which values to choose (see Table 1). This could be very helpful for authors who start using RQA measures for sleep apnea detection through HRV analysis. In particular, according to our results, we suggest using dimensions 7–8 and delays 4–5, for the embedding, and the FAN method with 5% of neighbors. In fact, a wider range of dimensions, from 5 to 9, could be considered, as the system performs similar with these values. Second, we have combined the commonly used RQA measures with other RQA features that, to the best of our knowledge, no author has used before to discriminate between apnea and nonapnea minutes.

Although the main conclusions reached are independent of the database, in the results analysis we could see several disparities that may be a result of discrepancies in apnea scoring in different sleep laboratories.

### Reference values for RQA parameters

Defining values that yield the best results allows us to fulfill one of the main objectives of the article: to further our understanding of sleep apnea characterization based on cardiac rate, and to infer information about the underlying physiological processs. According to the dimensions obtained after the results analysis, 7–8, we can see that dimensions proposed by Zbilut et al [55] for biological systems, namely 10, or by Webber and Zbilut [63], from 10 to 20, were very high and not suitable for all biological systems. As the dimension corresponds to the number of variables expected to influence the system under study, it is worth analyzing the possible cardiorespiratory variables that directly influence the HR control system. Nguyen et al. [13] were the first authors who related the dimension to the cardiorespiratory variables. In their work they propose 6 for the dimension, and the 6 variables they refer to are the following: cardiac output [88], blood pressure [89], respiratory rate [90], SpO2, cardiac repolarization (QT interval) [91] and central venous return [92]. Nevertheless, our results suggest that the underlying system is affected by 7 to 8 variables. Thus, it is important to emphasise the complex interactions that take place in physiological systems. For instance, Riedl et al. [93], based on Granger causality [94], studied the dynamical changes produced in pregnant women suffering from pre-eclampsia. They concentrated on the analysis of the coupling between respiration, systolic and diastolic blood pressure, and heart rate. According to their results, they referred to the baroreflex sensitivity as one of the essential variables to be considered for diagnostic puposes. As, according to the findings of Carlson et al. [95], OSA patients show an impaired baroreflex sensitivity, it seems reasonable to include this variable in the previous list. On the other hand, as blood pressure itself contains two different types of information, namely systolic and diastolic blood pressure, we could consider that the number of variables agrees with the dimension proposed in this work. Moreover, further studies would be necessary to define the complexity of the system in terms of structural and functional relationships between the variables. It is also worth noting that the HRV is not only influenced by apnea, but also by many additional factors, such as sleep stages, other diseases or medication, that may mask the CVHR pattern [26].

The choice of the delay is trickier (see Figs 3 and 4) as outcomes change significantly depending on its value. According to the delay reconstruction theorem, practically any delay should be appropriate for the embedding [96]. Grassberger et al. [97] also stated that the delay is a noncritical parameter and hence many delays should be suitable for the same system. But in practice, we can infer from our results that not any delay is convenient for the embedding. Values around 4–5 seem to be suitable to construct state vectors that are not autocorrelated. According to Fig 3C, the delay value chosen by Nguyen et al. [13] for the Physionet database, namely 10, seems to be too high. However, they were able to improve the results obtained with previous recurrence analysis-based approaches. They argued that the main reason, therefore, was a different choice of RP parameters that effectively exploited the difference in nonlinear and nonstationary dynamical information of HRV data during normal and apneic breathing. In our opinion, this rise may have been partially due to the introduction of the FAN Method and partially due to the sophisticated classification process. They used support vector machines (SVM) and neural networks (NN), and a soft decision fusion rule to combine the results of the classifiers. It is worth noting that our work, using only an LDA classifier and 9 features instead of 33, performs better. However, they only included the commonly used RQA measures.

As we have stated in the previous reasoning, and in accordance with other authors, such as Ngamga et al. [79], the selection of proper embedding parameters becomes complicated due to the highly non-stationarity of cardiovascular dynamics. In any case, we recommend embedding when RQA is applied to HRV in sleep apnea, because results are consistently better (see Figs 3 and 4) than those obtained without embedding for a wide range of dimensions and delays, especially in the FAN method.

The FAN method is more suitable for the distance threshold, for a variety of reasons: both AUCs and accuracies are better than in the Fixed Distance Method, for both databases we find similar values for dimension and delay that yield good results, and 5% of neighbours seems to be an adequate value for most cases. Moreover, this implies that the number of neighbours is important [48]. In our opinion, this result is related to the dynamic nature of the state space that represents the underlying system. This dynamic behaviour implies that attractors and couplings between the different variables that affect the system, also evolve with time. In this regard, the FAN Method could be considered as a way to ‘normalize’ the state space. The fact that the FAN Method is more convenient in this context agrees with other authors’ findings. Webber and Marwan stated that, although using a fixed radius is the most commonly used neighbourhood, the FAN Method is more suitable for nonstationary data, such as HRV data, as it allows an analysis based on comparable recurrence structures [98]. On the other hand, Nguyen et al. were the first authors who considered FAN better for HRV analysis in the context of sleep apnea, since it does not require attractors to be of a similar volume for the comparison of state-space behaviours [13].

In summary, there are two crucial conclusions, drawn from the results obtained in the data analysis. First, there is a practical interdependence between the different parameters involved in the RQA approach, namely the embedding (dimension and delay) and the distance parameters. Second, the system performance is completely dependent on the parameter selection. Moreover, we consider the exhaustive exploratory analysis performed in this article of special interest for authors who want to apply RQA to HRV analysis in the context of sleep apnea, since they could apply the values proposed for the RQA parameters as reference values.

### Physiological interpretation of selected features

According to the results, vertical and diagonal measures in RPs carry fundamental information for classification purposes, regardless of the database, and of the method used to define the distance threshold. This coincides with the outcomes of Marwan et al. [56] when applying RQA to HRV data. However, in the context of sleep apnea, Maier and Dickhaus [34] stated that the appereance of the RPs for apnea and nonapnea minutes (Figs 6 and 7) suggested the importance of the diagonal structures to detect apneic events. In our opinion, the reason why they obtained poor outcomes using RQA measures could be that they only considered the features related to diagonal ones. In this way they left out other measures that contribute a great deal to the system performance. Besides, they defined l_min_ = 4, the minimal length to consider a diagonal line, instead of 2, a more commonly used value for this parameter. Le et al. [11] and Karandikar et al. [12] pointed out in their works the importance of the vertical lines. Using the Fixed Distance Method applied to the Physionet database, they indicated that the most sensitive RQA feature was Vmax, followed by LAM. However, in our opinion, both types of measures, vertical and diagonal, are crucial to extract from the RPs as much information as possible, and therefore, should be included in any study related to sleep apnea where RQA is applied to HRV. The fact that vertical RQA measures are very sensitive to the embedding makes the selection of dimension and delay an especially important issue [56].

Beyond the importance of vertical and diagonal structures to detect OSA events, it is necessary to analyse the values obtained for the most relevant measures to interpret their physiological significance. According to the results, LAM and Vmax, turn out to have a relevant role in the apnea minutes discrimination. The values obtained for LAM and Vmax (see Figs 9 and 10) are always higher for apnea minutes than for nonapnea minutes. This is due to the occurrence of laminar states, i.e. states that do not change or change very slowly during apnea. LAM gives information about the presence of laminar phases, and Vmax about their duration [45]. LAM and Vmax are inversely proportional to the system complexity. This means that low LAM and Vmax values, found in nonapnea minutes, imply high complexity in the system dynamics, because the system stays briefly in a state similar to the previously occurring one [41]. This behavior can be related to the operation of the cardiovascular regulatory sytem, which is influenced by several factors. In healthy subjects, the cardiovascular system reacts inmediately to system stimuli, thus decreasing the time the organism stays in the same or similar state. However, under pathological circumstances, the control system simplifies and the recurrences to similar states increase [52]. This suggests a loss of complexity and more regularity in the system in the presence of apnea. These results are in line with other authors’ findings, not only in the context of the cardiovascular system: Mendez et al. [6] refer to the HRV loss of complexity in sleep apnea, Javorka et al. [41] point out the complexity loss and simplification of heart rate dynamics in patients with diabetes mellitus, and Subramaniyam et al. [99] report an increasing degree of structural complexity in the EEG of normal subjects compared to those of patients with epilepsy. Several authors have attempted to explain the causes of this behavior [27,95]. Trzebski et al. [27] studied the nonlinear dynamics of the cardiovascular systems in humans exposed to repetitive voluntary apneas, modeling OSA, and their results also suggest a reduction in the complexity of the cardiovascular control system. One of the reasons they give is the attenuation or inhibition of the arterial baroreflex by chemoreceptor stimulation. In fact, Carlson et al. [95] already stated that OSA patients show an impaired baroreflex sensitivity.

DET and Lmax also increase their values during apnea minutes because the diagonal structures, due to the RSA, are not as evident as those that appear in apnea phases related to the CVHR (see Figs 6 and 7). Higher values during apnea minutes indicate higher predictability and regularity of the system dynamics over time, as stated for Vmax and LAM [41,45]. For a single beat, an increase in DET or Lmax means a higher probability of remaining in the same state as the previous one.

The clustering coefficient gives the probability that two neighbours of any state are also neighbours [72]. Hence, in a periodic system, this measure would take its largest value (CC = 1). Therefore, assuming the increase of diagonal structures in the RPs during apnea events (see Figs 6 and 7), we find higher values for apnea minutes. This measure is particularly important because it has no direct counterpart in RQA [72]. Moreover, it highlights the importance of the quantification of the RP structure, and the topology of the phase space.

In general, we can find the same behavior in the remaining features: higher values for apnea minutes. This fact implies lower HRV, simplification of heart rate dynamics and greater predictability and, ultimately, pathological conditions [41,52].

### Limitations of the proposed method

There are two parameters whose effects were not assessed in our experiments, namely the norm (Euclidean, minnorm and maxnorm) and the Theiler window. We always used the Euclidean norm, as Marwan et al. [45] pointed out that there are small differences between the Euclidean norm and the maxnorm. The minnorm is rarely used. As far as the Theiler window is concerned, it was suggested by Theiler in 1986 [100], because it is common to find small distances between points in the reconstructed phase space that are close in time. The Theiler window is usually set to the value of the time delay *τ* or to (*m*-1)*τ*, according to Javorka et al. [53] or to Marwan et al. [45], respectively. Thus, only points that are farther than *τ* from the diagonal are taken into account for the evaluation of the RQA measures [41]. As in most studies, no Theiler window was used in our experiments. These two parameters are in general not as critical as those evaluated in the current work. Nevertheless, we will consider their assessment in future work to study the possible effects on the results obtained for sleep apnea detection.

On the other hand, some limitations related to the databases need to be pointed out in this study. One of the databases employed for the analysis was the widely used Physionet database, which presents some drawbacks, e. g. a restricted number of subjects, whose age ranges from 27 to 63, or a small number of women included in the dataset (in groups A and B only one each). The latter is especially important as, according to recent studies, there are potential gender differences in HRV sleep apnea information [15]. In future studies, this consideration should be taken into account for a differentiated learning and validation process. Moreover, it would be desirable to include in the database apneic patients with various cardiovascular disorders, that would probably have an impact on the system performance. Therefore, for a clinical validation of the proposed approach, a larger database, including older participants, a higher number of women, as well as cardiac patients, would be necessary. As far as the HuGCDN2014 is concerned, the lack of subjects representing mild and moderate OSA patients in the database is also a limitation that should be considered.

Finally, it should be also pointed out that the difference in the sample frequency used in both databases for the single-lead ECG signal, namely 100 (Physionet) and 200 Hz (HuGCDN2014) could influence the measured distance between consecutive R-peaks.

### Comparison with prior work

The performance of the OSA classification approach proposed in this article is compared with existing literature. Table 10 shows a selection of the most representative methods that employ the widely used Physionet database with the results obtained for per-segment classification as, for meaningful comparison, results obtained from the same database have to be compared.

**Table 10:** Comparison of per-segment OSA detection results on Physionet database.

Method	Year	N° of recordings	N° of features	AUC	Acc(%)
Spectral features and LDA [8]	2000	70	30	-	88.31
Temporal and spectral RR and EDR features and LDA [101]	2003	70	88	-	90
Sample entropy, spectral features [9]	2007	70	6	-	72.9
Temporal and spectral features from RR and QRS area and kNN [6]	2009	50	10	-	88
WA and QDA [7]	2010	50	10	-	89.07
RQA of HRV and EDR, and Autoeural model [12]	2013	70	21	-	88.06
RQA and soft decision fusion rule (SVM and NN) [13]	2014	70	72	-	85.26
			33		84.19
Principal components of QRS and orthogonal subspace projections (LS-SVM) [14]	2015	70	6	0.88	84.74
Hermite basis functions and LS-SVM [17]	2016	70	5	0.83	83.8
Heterogeneous recurrence analysis [18]	2016	35	11	0.91	82.5
RQA (FAN) and LDA (Proposed approach)	2017	70	9	0.9253	
			11		86.33

Unlike other comparative studies, particular relevance is given to the number of features and the number of recordings used in the experiments. As shown in Table 10, Mendez et al. [6–7] reach high accuracies. However, they rule out 20 recordings that do not satisfy certain criteria of data quality. So, their method requires high-quality datasets, that are not normally available, as physiological signals are, by nature, noisy. In this regard, our method is more robust because it does not require a preselection of high quality data.

Other notable outcomes are those reached by Schrader et al. [8], de Chazal et al. [101], Karandikar et al. [12] and Nguyen et al. [13]. In all these studies, the main weakness is the high-dimension feature space, over 20. Cheng et al. [18] compare the performance obtained by classical RQA against heterogeneous RQA. In the latter approach, they propose segmenting the state space into a hierarchical structure of local recurrence regions, i.e. in addition to the RQA parameters (delay, dimension and distance threshold), they have to determine the optimal number of subregions. Despite the increased method complexity, their results are worse than ours. Moreover, there are authors, like Varon et al. [14] that derive two signals from the ECG, RR and EDR. However, in our approach, only the RR interval series is obtained from the ECG, thus simplifying the preprocessing stage.

From Table 10 we can conclude that the proposed method outperforms those proposed in the most recent literature. In particular, the results obtained are better than the best ones reached so far using RQA on sleep apnea [13]. So, we can consider our outcomes very promising. In this regard, it is important to highlight that, although other authors have questioned the utility of RQA to yield additional insight into sleep apnea recognition from HRV [34], the results obtained in this article demonstrate that applying adequate values for the RQA parameters, and including other RQA measures in addition to the classic ones, yield better results than other previous approaches.

In summary, outcomes indicate that the use of single-lead ECGs can perform well in the detection of sleep apnea events. Nevertheless, more effort should be done to construct a model and to define the most valuable features for a better understanding of the physiological phenomena underlying sleep apnea.

## Conclusions

This article presents a methodology for the automatic detection of sleep apnea events from single–lead ECG. It is based on nonlinear cardiorespiratory dynamics and the contributions to the state of the art are twofold. On the one hand, we focused on finding reference values for the different parameters implied in the RQA approach, namely dimension, delay and threshold distance, when RQA is applied to HRV in the context of sleep apnea as, until now, there is no agreement about which values to choose. Therefore, two different databases were introduced in order to give results a more generalizable character. In this respect, we have concluded, after intensive computational analysis of recurrence, that working with dimensions around 7–8 and delays about 4–5, together with the FAN method with 5% of neighbours, yield the best results.

Second, we have combined the commonly used RQA measures with other RQA features that, to the best of our knowledge, no author has used before to discriminate between apnea and nonapnea minutes. In this respect, we concluded that the newly used features, especially the clustering coefficient (Clust), the entropy of the white vertical lines (ENTW) and the maximal recurrence time (RTmax), contribute valuable information about the presence or absence of breathing pauses during sleep. So, these features can be considered especially interesting for characterization purposes.

As far as the results are concerned, our system outperforms, using a relatively small set of features, other methods reported in the most recent literature for fully automated algorithms. In particular, the best results in Physionet are AUC = 0.93 and Acc = 86.33%, and in HuGCDN2014, AUC = 0.86 and Acc = 84.18%.

In summary, we have proposed a method that performs well in the detection of sleep apnea events and can help us further our understanding of the underlying process from a cardiac rate point of view. The fact that we use only single-lead ECG signals and a classifier that shows a good performance/complexity ratio encourages the development of home-based OSA screening devices.
